# Public Health and Economic Benefits of Influenza Vaccination of the Population Aged 50 to 59 Years without Risk Factors for Influenza Complications in Mexico: A Cross-Sectional Epidemiological Study

**DOI:** 10.3390/vaccines9030188

**Published:** 2021-02-24

**Authors:** Miguel Betancourt-Cravioto, Jorge Abelardo Falcón-Lezama, Rodrigo Saucedo-Martínez, Myrna María Alfaro-Cortés, Roberto Tapia-Conyer

**Affiliations:** 1 Vaccinology Section, Sociedad Mexicana de Salud Pública, Mexico City 11590, Mexico; betancom70@gmail.com (M.B.-C.); jfalcon@fundacioncarlosslim.org (J.A.F.-L.); rosaucedo@gmail.com (R.S.-M.); myrna.alfaro.fcs@gmail.com (M.M.A.-C.); 2 School of Medicine, Universidad Nacional Autónoma de México, Mexico City 04510, Mexico

**Keywords:** influenza, vaccination, adult-aged population, cost effectiveness, Mexico, burden of disease, middle-income countries

## Abstract

The Mexican influenza vaccination program does not include a recommendation for people aged 50–59 years without risk factors for influenza complications, and there are limited data regarding the cost-effectiveness of vaccinating this population. To explore the clinical and economic effects of including this population in the vaccination schedule, we performed a cross-sectional epidemiological study using records (2009–2018) from Mexico’s Influenza Surveillance System (SISVEFLU), death records (2010–2015) from the National Mortality Epidemiological and Statistical System, and discharge and hospitalization records (2010–2015) from the Automated Hospital Discharge System databases. A 1-year decision-analytic model was used to assess cost-effectiveness through a decision-tree based on data from SISVEFLU. The primary outcome was influenza cases avoided; with associated influenza-related events as secondary outcomes. Including the population aged 50–59 years without risk factors in Mexico’s influenza immunization program would have resulted in 199,500 fewer cases; 67,008 fewer outpatient consultations; 33,024 fewer emergency room consultations; 33,091 fewer hospitalizations; 12 fewer deaths. These reductions equate to a substantial public health benefit as well as an economic benefit; yielding net savings of 49.8 million US dollars over a typical influenza season. Expansion of the current Mexican vaccination schedule to include these people would be a cost-saving and dominant strategy.

## 1. Introduction

Every year, influenza affects millions of people of all age groups, which has substantial public health and economic impacts [1]. It is estimated that there are between 3 and 5 million cases of severe disease each year and around 0.5 million deaths globally [2]. In Latin America, the annual incidence of influenza-like illness (ILI) ranges between 4.7% and 15.4% [3].

Although safe and effective influenza vaccines have been available for more than 80 years [4,5,6], the constant genetic shifts and drifts of the virus remain the most relevant challenge for disease control. Nevertheless, implementation of influenza immunization campaigns throughout the world have led to decreases in both mortality and morbidity [7,8]. Vaccination in most age groups, including adults, is considered highly cost-effective [9,10].

World Health Organization (WHO) recommends the annual administration of the influenza vaccine for all individuals ≥6 months of age [11]. National recommendations vary significantly between countries and focus mostly on children between 6 months and 5 years of age and the population ≥60 years of age, leaving out the population aged 50 to 59 years. This segment of the overall population, from a societal perspective, is relevant as it is a sizeable proportion of the economically active population, and the impact of influenza on productivity losses in this age group is important.

The United States of America, Panama, and Chile are the only countries in the Americas to recommend universal influenza vaccination for the population aged 50 to 59 years [12,13,14]. Throughout the rest of the world, 10 other countries (Austria, Estonia, Poland, Malta, Slovenia, Bahrain, Libya, Ireland, Laos, and the Marshall Islands) also recommend universal application of the influenza vaccine, including the population aged 50 to 59 years [15,16,17].

In Mexico, the current national vaccination schedule recommends yearly immunization in several target groups: children aged 6 to 59 months, adults aged ≥60 years, pregnant women, health professionals, and population aged 5 to 59 years with risk factors for influenza complications (diabetes, hypertension, obesity, chronic kidney disease and asthma, among others). These target groups are eligible to receive the vaccine free of charge at any public health facility during the influenza immunization season (October to February). It should be noted that Mexico’s public health system provides different levels of healthcare coverage according to employment status (private company workers, government workers, and unemployed). Healthcare services are provided free of charge to approximately 85% of the population [18]. However, the vaccination program for influenza (and for 13 other vaccines) is universal and is provided at no cost to eligible individuals, regardless of their healthcare coverage. Currently, vaccination of adults aged 50 to 59 years without risk factors for influenza complications is not considered [19].

Studies have shown that the cost-effectiveness of influenza vaccination varies depending on the age-groups targeted [20]. De Waure et al. assessed the economic benefits of influenza vaccination and found that most studies included in their review analyzed either the cost-effectiveness or the cost–benefit of vaccination, with other studies reporting this strategy to be cost-saving [21]. In particular, for the population aged 50 to 59 years, a literature review by Dabestani et al. showed that the cost-effectiveness of influenza vaccination ranged from USD 8000 to 39,000 per quality-adjusted life year [22].

In this study, we analyzed epidemiologic and disease burden data from influenza seasons 2009–2010 to 2017–2018 (Northern Hemisphere) to calculate the health and economic benefits of implementing influenza vaccination in the population aged 50 to 59 years without risk factors for influenza complications for a typical season. For influenza vaccination in Mexico, the only available vaccine is a seasonal hemispheric vaccine, which is the one considered for the current analysis. Using a decision-analytic model, we estimated the cost-effectiveness of this intervention, considering the reduction in the number of influenza cases (including deaths associated with influenza) as the primary health outcome from the third-party payer and societal perspective, and the influenza-related events associated with this reduction as secondary health outcomes. The timeframe of the analysis was 1 year to reflect the seasonality of the disease.

## 2. Materials and Methods

We performed a cross-sectional epidemiological study using the following databases: (1) an anonymized database from Mexico’s Influenza Surveillance System (SISVEFLU) obtained upon written request from Mexico’s General Directorate of Epidemiology, which included all influenza records from November 2009 to October 2018; (2) the mortality database of the National Mortality Epidemiological and Statistical System (SEED) for the period of 2010–2015 (data from 2009 and 2016–2018 were not available) [23]; and (3) discharge and hospitalization data obtained from the Automated Hospital Discharge System (SAEH) for the period 2010–2016 (data from 2009 and 2017–2018 were not available) [24]. For both SEED and SAEH, cases were selected following the International Statistical Classification of Diseases and Related Health Problems, 10th revision [25]. Data for projections of the Mexican population for the study period were obtained from the National Population Council database [26].

SISVEFLU is an automated system (since 2009) that uses a network of monitoring health care centers and hospitals distributed across the country to provide information on trends in circulating viral strains and the occurrence of severe cases [27]. Cases are initially classified as either ILI or severe acute respiratory infection (SARI). Polymerase chain reaction (PCR) is used for diagnosis confirmation at FluNet collaborating facilities of the National Network of Public Health Laboratories (Red Nacional de Laboratorios de Salud Pública) [28]. For this study, we used the ILI and SARI case definitions used in the current Mexican influenza surveillance guidelines [27].

### 2.1. Case Classifications

**Figure 1:**
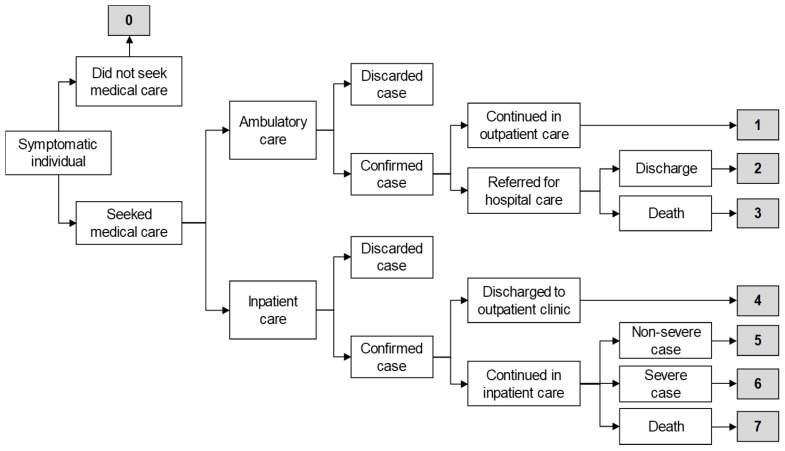
Decision tree for case classification [29]. For details see Section 2.1.

Based on data from SISVEFLU, eight health outcome scenarios were considered for estimating the costs of influenza cases (Figure 1). We took into consideration whether an individual sought medical care or not (scenario 0), ambulatory care (scenarios 1 through 3), or hospital care (scenarios 4 through 7):

1. Scenario 0: Symptomatic individual did not seek medical care, self-medicated with over-the-counter drugs, and had a complete recovery.
2. Scenario 1: Symptomatic individual visited an outpatient clinic, had a positive PCR result for influenza, was managed only in ambulatory care, and had a complete recovery.
3. Scenario 2: Symptomatic individual visited an outpatient clinic, had a positive PCR result for influenza and was referred for hospital care due to severity, had a complete recovery, and was discharged.
4. Scenario 3: Symptomatic individual visited an outpatient clinic, had a positive PCR result for influenza and was referred for hospital care due to severity, and died.
5. Scenario 4: Symptomatic individual visited a hospital emergency room (ER), had a positive PCR result for influenza, was discharged to an outpatient clinic for follow-up, and had a complete recovery.
6. Scenario 5: Symptomatic individual visited a hospital ER, had a positive PCR result for influenza, was admitted to hospital for follow-up with non-severe clinical status, and had a complete recovery.
7. Scenario 6: Symptomatic individual visited a hospital ER, had a positive PCR result for influenza, was admitted for follow-up with severe clinical status, and had a complete recovery.
8. Scenario 7: Symptomatic individual visited a hospital ER, had a positive PCR result for influenza, was admitted to hospital for follow-up, and died.

Table 1 details the assumptions made for clinical management of cases for each scenario; they were defined according to the National Clinical Guidelines for the Prevention, Diagnosis, and Treatment of Seasonal Influenza in Mexico, as follows [30]:

### 2.2. Laboratory Diagnosis

Confirmation of influenza is based on real-time PCR results. In ambulatory care, sample collection for confirmation is required for only 10% of cases, whereas 100% of cases are mandated for sample collection for confirmation of influenza in inpatient care [28]. Throat swab culture is recommended for cases with suspected bacterial coinfection.

### 2.3. Medical Consultations

For the purposes of this study, it was assumed that cases detected and managed in outpatient clinics (scenario 1) required two medical consultations; the first for clinical diagnosis and prescription of treatment, and the second to confirm complete recovery. Patients admitted to hospital via an outpatient clinic (scenario 2) required one ambulatory consultation, and two specialty consultations, the first to begin treatment at the hospital and the second at discharge. For scenario 3 (admission to hospital via an outpatient clinic that results in death), we considered one initial ambulatory consultation, where clinical diagnosis and referral occurred, and three specialty consultations at the hospital. An emergency consultation was considered mandatory for all hospital-managed patients admitted through the ER (scenarios 4–7). In these scenarios, patients had one, two, three, and four medical consultations in scenarios 4, 5, 6, and 7, respectively, assuming a proportional increase in the number of medical consultations with the disease severity.

### 2.4. Drugs

For individuals not requesting medical care (scenario 0) and only requiring over-the-counter drugs, we assumed the use of amantadine for influenza treatment, and paracetamol for management of general symptoms. For all confirmed cases, either ambulatory or inpatient care, we assumed the prescription of oseltamivir for influenza treatment, and paracetamol. For antibiotic treatment, we assumed the use of ceftriaxone in patients with a bacterial coinfection (scenarios 2, 3, 5, 6, and 7).

### 2.5. Days of Hospitalization

For patients admitted via an outpatient clinic who were referred for hospitalization and later discharged (scenario 2), we assumed a 1-day hospital stay, mostly for monitoring of symptoms and clinical evolution. For patients admitted via an outpatient clinic who were referred for hospitalization that resulted in death (scenario 3), we considered the average hospital stay (8.3 days) for patients aged 50 to 59 years with influenza, according to SAEH [24]. Very importantly, we assumed that a hospital stay is provided independently of the admitting area or laboratory confirmation of the case, and that those unit costs are already taken into account. Patients admitted to hospital for observation via the ER who were discharged for follow-up at an outpatient clinic (scenario 4) were considered to have had a 2-day hospital stay to monitor their progress until discharge. Patients with non-severe cases admitted via the ER for medical care (scenario 5) were considered to have the average 8.3-day hospital stay. For patients with severe cases admitted to hospital via the ER (scenario 6), we assumed a 50% longer hospital stay (12.45 days) than patients with non-severe cases. Finally, for patients admitted via the ER whose outcome was death (scenario 7), we assumed the average hospital stay of 8.3 days.

### 2.6. Days of Medical Disability Leave

Days of medical leave were estimated based on the guidelines of the Mexican Institute of Social Security. For patients diagnosed in outpatient clinics without hospital admission (scenario 1), a 3-day medical leave was assumed. For patients diagnosed in outpatient clinics with a hospital referral (scenario 2), a 7-day medical leave was assumed after a 1-day hospital stay, for a total of 8 days of leave. For patients admitted via the ER and later discharged for follow-up at an outpatient clinic (scenario 4), a 3-day medical leave was assumed after a 2-day hospital stay, for a total of 5 days of leave. For those admitted via the ER and hospitalized as non-severe cases (scenario 5), a 7-day medical leave was assumed after an 8-day hospital stay, for a total of 15 days of absence. For patients admitted via the ER and hospitalized as severe cases (scenario 6), a 14-day medical leave was assumed after a 12-day hospital stay, for a total of 26 days of absence [31].

### 2.7. Years of Life Lost

Following Mexico’s current life expectancy, the ages of 73 and 78 years are considered for males and females, respectively, and these ages were later weighed according to the population distribution by sex and discounted using a 5% rate following WHO recommendations [32].

### 2.8. National Estimates of Influenza Cases

SISVEFLU cases are recorded in monitoring facilities without geographic or population representativeness (e.g., clinics were selected based on their capacity to diagnose influenza, or their capacity to concentrate a large volume of influenza cases). Therefore, data cannot be extrapolated directly to population-wide estimates. To overcome this limitation, the total number of influenza cases in Mexico was estimated by applying reported values of influenza incidence published by the Centers for Disease Control and Prevention (CDC), for the United States for each season and age group [33], to the Mexican population structure (an in-depth analysis is provided in Text S1 and Tables S1.1–S1.2) [26]. This method was based on the assumption that epidemiological factors influencing transmission, such as circulating viruses, epidemic behavior, geographic proximity, and vaccine efficacy, are similar in the two countries.

National estimated cases were then allocated into the different scenarios considering the probability of occurrence: (1) not demanding medical care (scenario 0) [34], (2) demanding medical care with complete recovery (scenarios 1, 2, 4, 5 and 6) [SISVEFLU], and (3) demanding medical care that resulted in death (scenarios 3 and 7) [SISVEFLU and validated in the SEED database]. Further details of the method used to estimate national cases are available in Text S2 and Tables S2.1–S2.4.

### 2.9. Estimation of the Population Without Risk Factors for Influenza Complications

Following the methodology of Ruiz-Palacios et al. [35], we estimated that 47.33% of the population aged 50 to 59 years has at least one risk factor for influenza complications, consistent with data from SISVEFLU where 48.73% of patients were reported to have at least one risk factor for influenza complications. Therefore, the remaining 52.67% of the population was considered as being without risk factors and, thus, would not be covered by the current vaccination schedule; details of the estimation of the population with risk factors for influenza complications are provided in Tables S3.1–S3.2 (estimations were corrected for double counting of patients with risk factors based on published literature [36,37,38,39,40]).

### 2.10. Unit Costs for the Estimation of Economic Burden of Influenza

To estimate direct medical costs, we considered public unit costs of each of the institutions that comprise the Mexican Health System, and then weighted them by the proportion of the population affiliated in each institution for the influenza seasons from 2009–2010 to 2018–2019 (details are provided in Tables S4.1–S4.4). Regarding indirect costs, absenteeism due to influenza was estimated using the average daily wage of an individual (obtained from the 2018 National Survey of Household Income and Expenditure) [41]. Finally, indirect costs associated with premature deaths were projected depending on the age of an individual at the time of death, and they were discounted using the recommended 5% rate.

Costs were originally obtained in Mexican pesos (MXN) and later converted to 2018 constant prices using the National Consumer Price Index published by Mexico’s National Bureau of Statics and Geography. Data are presented in US dollars (USD) using the average exchange rate published in the Official Federal Gazette between January 2019 and August 2019 (USD 1 = MXN 19.2155).

### 2.11. Costs of Vaccination

Mexico currently uses the trivalent inactivated influenza vaccine in the national immunization program. The price per dose of influenza vaccine was obtained from Mexico’s Ministry of Health for 2018 (MXN 57.68, USD 3.00), whereas the cost of administration (MXN 4.54, USD 0.24), and the cost of transportation and storage (MXN 0.55, USD 0.03) were obtained from Gutierrez and Bertozzi’s study and converted to 2019 prices [42].

### 2.12. Vaccine Coverage and Effectiveness

Effectiveness of the influenza vaccine was set at 50%, which is the average effectiveness in the Northern Hemisphere of the Americas as published elsewhere for influenza seasons from 2009–2010 to 2017–2018 (Table S5). Coverage was defined at 50%, assuming a slow uptake in the first annual implementation of a vaccination campaign.

### 2.13. Influenza Outcomes

The primary health outcome for this study was influenza cases avoided (including deaths attributable to influenza), and their influenza-related health events such as reductions in outpatient consultations, specialty consultations, ER consultations, hospitalizations, and lost working days.

### 2.14. Sensitivity Analysis

We performed a sensitivity analysis to assess whether the cost-effectiveness of universally immunizing population aged 50 to 59 years was sustained in either a conservative or an optimistic scenario, considering changes to the base case scenario in both vaccination coverage and vaccination effectiveness. For the conservative scenario, we assumed a vaccination coverage of 30% and a vaccination effectiveness of 19%, which is the lowest effectiveness reported for any influenza vaccine used from seasons 2009–2010 through to 2017–2018 in the Northern Hemisphere of the Americas. For the optimistic scenario, we assumed a vaccination coverage of 70% and a vaccination effectiveness of 68%, which was the highest effectiveness reported in the same period and region.

## 3. Results

### 3.1. Epidemiology

From November 2009 to October 2018, Mexico’s SISVEFLU system recorded 50,900 laboratory confirmed cases out of 390,862 probable ILI/SARI cases for all ages (13.02% positivity proportion). The number of probable ILI/SARI cases in the population aged 50 to 59 years was 36,647, of which 5725 (15.62%) were confirmed. From these, 2935 confirmed cases reported not having risk factors for influenza complications (Table 2).

The season with the highest record of confirmed cases was 2013–2014 with a total of 623 cases (601 influenza A cases and 22 influenza B cases) reported. Influenza A virus was isolated in 86.06% of the cases. This variant accounted for 95.38% of deaths. Cases, clinical presentation, viral types, and deaths were consistent between seasons.

As for lethality (Table 3), the overall rate was 8.86% (260/2935). However, there were important variations depending on the infecting virus. The highest lethality was recorded for A H1N1 (13.78%), followed by A not subtyped (3.93%), B undetermined lineage (3.08%), B Yamagata (3.05%), and A H3N2 (2.11%). No cases of lethality were reported for B Victoria in the population aged 50 to 59 years without risk factors for influenza complications.

**Figure 2:**
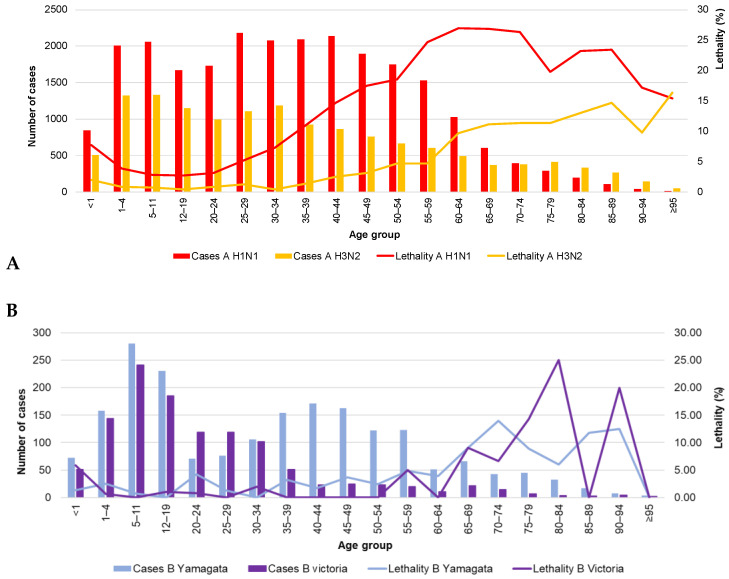
Cases and lethality by age group (2009–2018) and virus type. (**A**) influenza A; (**B**) influenza B. The data used in this figure were obtained from Mexico’s influenza surveillance system, SISVEFLU.

**Figure 3:**
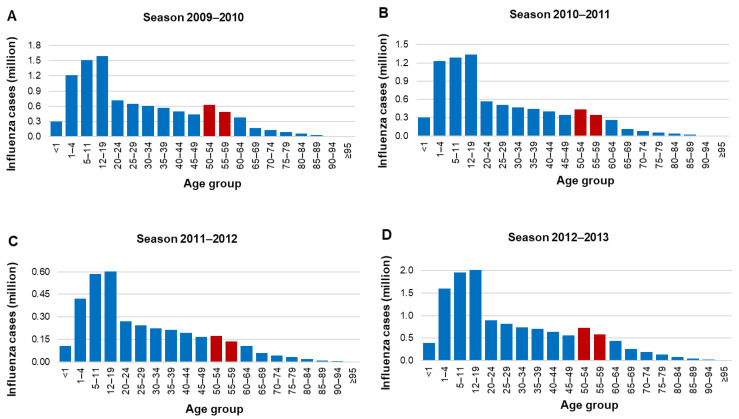

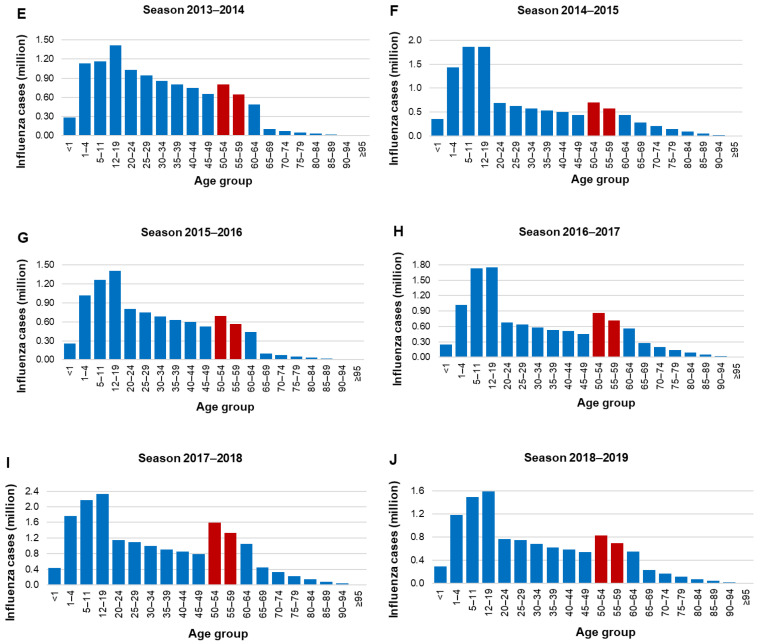
Estimated influenza cases by age group and season. (**A**) 2009–2010 season, (**B**) 2010–2011 season, (**C**) 2011–2012 season, (**D**) 2012–2013 season, (**E**) 2013–2014 season, (**F**) 2014–2015 season, (**G**) 2015–2016 season, (**H**) 2016–2017 season, (**I**) 2017–2018 season, and (**J**) 2018–2019 season. Each graph is set to a different scale; all seasons are reported in million cases.

In general, there seems to be an age-dependent, negative relationship between the number of cases and lethality caused by influenza A H1N1 in the Mexican population. Younger age groups contribute an important number of cases but have low lethality rates, while older age groups register the highest lethality but present proportionally fewer cases (Figure 2A). This phenomenon is not observed for influenza B (Figure 2B). As seen in Figure 3A–J, the number of cases in the population aged 50 to 59 years is relatively high compared with the downward trend in the age groups of 20 to 24 years through 40 to 49 years. Thus, expanding influenza immunization in this age group to include those without risk factors would potentially yield enormous health benefits.

### 3.2. Hospital Discharges

Table 4 shows total hospital discharges for which influenza was the main diagnosis (2010–2016 period) in the population aged 50 to 59 years.

Of individuals aged 50 to 59 years, there were 1065 discharges from hospital during the study period, accounting for 8831 bed-days, with an average of 8.3 bed-days per hospitalized patient. The most frequent diagnosis was influenza with other respiratory manifestations, virus not identified (J111), followed by influenza with pneumonia, virus not identified (J110).

### 3.3. Mortality

Table 5 shows mortality by season for all age groups. Overall mortality was low (0.3/100,000 individuals). The population aged 50 to 59 years had the second highest mortality, just below that observed for the <1 year and ≥60 years of age populations.

### 3.4. Cost-Effectiveness

The estimated expansion of the current influenza immunization program to include vaccination of the population aged 50 to 59 years without risk factors for influenza complications over the 2018–2019 season, with an assumed 50% coverage and 50% effectiveness, resulted in a reduction of 199,500 cases (Table S6.1). Of the total cases averted, 66.6% were from people who did not seek medical care (scenario 0), 16.8% from patients who sought ambulatory care (scenarios 1 and 2), and 16.6% from patients who sought inpatient care (scenarios 4, 5, and 6). In addition, this reduction of cases led to 12.1 fewer deaths (Table S6.2).

Overall, this reduction of cases would be associated with 67,008 fewer outpatient consultations, 53,790 specialty consultations, 33,024 ER consultations, and 33,091 hospitalizations (Table 6).

Without vaccination of the population aged 50 to 59 years without risk factors for influenza complications, the economic burden of influenza treatment would account for 241.27 million USD (Table 7), with the greatest burden on hospitalizations, which represent 69.1% of the total cost, and productivity loss (13.4% of the total). If we consider the distribution of treatment costs per scenario, cases that were treated through inpatient care (scenarios 4 to 7) would represent 92.9% of the total cost, whereas cases for which treatment began through ambulatory care (scenarios 1 to 3) would represent 6.5% of the total.

The economic cost of influenza treatment, after implementation of the influenza vaccination strategy, was USD 180.95 million (Table S6.3). In addition, vaccination of this group would cost USD 10.53 million (Table 8).

The number of estimated influenza cases avoided due to vaccination of the population aged 50 to 59 years without risk factors for influenza complications, in turn, would lead to a decrease in the economic burden for the Mexican health care system, yielding USD 49.80 million in net savings (Table 9). Of the total net savings, USD 40.74 million (81.8%) would be from direct costs, even when vaccination cost is considered. In addition, USD 9.06 million would be saved through decreases in absenteeism by preventing productivity loss and premature deaths. Therefore, this intervention is a cost-saving strategy that is dominant with respect to the current policy.

### 3.5. Sensitivity Analysis

Table 10 shows the sensitivity analysis results of estimated influenza cases averted after the immunization of the population aged 50 to 59 years. In the base case scenario (vaccination coverage: 50%; vaccination effectiveness: 50%), 199,500 cases could be averted, whereas in the conservative (vaccination coverage: 30%; vaccination effectiveness: 19%) and optimistic (vaccination coverage: 70%; vaccination effectiveness, 68%) scenarios, the number of potential cases averted would be 45,493 and 379,840, respectively. Of note, 66.6% of the cases (regardless of whether the base, conservative, or optimistic scenario is used) would correspond to the population who do not seek medical care (scenario 0), 16.8% would be symptomatic individuals who seek ambulatory care (scenarios 1 and 2), and 16.6% would be those who seek inpatient care (scenarios 4 to 6). Even when a conservative scenario is used, a substantial number of influenza cases are estimated to be avoided (45,493) when the population aged 50 to 59 years without risk factors for influenza complications is included in the national vaccination schedule.

Table 11 shows the results of sensitivity analysis for the estimated influenza-related events avoided in the three scenarios. Sensitivity analysis using a conservative scenario would result in the avoidance of 35,077 consultations (outpatient, specialty, and emergency room), 7546 hospitalizations, and 2.8 deaths. In an optimistic scenario, where 70% of the population aged 50 to 59 years is vaccinated and the vaccine efficacy is 68%, we estimated 292,870 fewer consultations, 63,003 fewer hospitalizations, and 23.0 fewer deaths. The mortality avoided through vaccination in the optimistic scenario would represent around 5.5% of the average annual deaths attributable to influenza during the study period.

We assessed the economic benefits of each of the three scenarios (Table 12). As mentioned in the base case scenario, influenza immunization to the population aged 50 to 59 years would result in USD 49.80 million in savings; in a conservative scenario, influenza immunization would result in USD 7.44 million in savings, out of which 5.38 million (72.2% of the total) would be direct savings, and 2.07 million indirect savings. Savings from hospitalizations alone (USD 9.50 million) would more than cover the cost of immunizing this population (USD 6.32 million). Our sensitivity analysis indicates that even in a conservative scenario, a policy of immunizing the population aged 50 to 59 years without risk factors for influenza complications is a cost-saving intervention, making it a dominant strategy with respect to the current policy.

## 4. Discussion

In Mexico, there are currently 12.2 million individuals aged 50 to 59 years (9.71% of the population). This proportion is expected to increase in the next 20 years to about 15.2 million individuals (11.06% of the total population) (Figure S1). From an economic perspective, this age group is relevant as it is a sizeable proportion of the economically active population and the impact of influenza on productivity losses is an important consideration. From a public health perspective, it is also very significant as there are between 0.3 and 2.9 million influenza cases annually in this age group, representing an average of 11.24% of the total influenza cases in Mexico (Text S3, Tables S7.1–S7.3); over 20% of deaths due to influenza also occur in this age group. The population aged 50 to 59 years has the second highest lethality compared with any age group in the entire Mexican population, even when considering only the fraction without risk factors for influenza complications (8.86%); the ≥60 years age group has the highest lethality (Tables S7.2, S7.4–S7.5).

Current recommendations for influenza vaccination in Mexico do not include the 50 to 59 years age group [43] unless individuals have risk factors for influenza complications. The National Advisory Committee on Immunization has previously recommended the universal application of the seasonal influenza vaccine in this age group to the public health authorities [44,45]; however, the recommendation has not been implemented. When evaluating the universal inclusion of the population aged 50–59 years for influenza vaccination in the national vaccination schedule, influenza-related productivity loss is an important consideration as this age group is still active in the labor market. Additionally, this age group has longer hospital stays compared with the rest of the population (8.3 days vs. 6.1 days, respectively) [24]. It should also be considered that the cost of vaccination in Mexico is very competitive with respect to other countries [46], and is quite low when compared to the cost of influenza treatment, particularly hospitalization. The costs reported in our study are consistent with a recently published assessment of the economic impact of switching from a trivalent to a quadrivalent inactivated influenza vaccine in Mexico [35].

Our study shows that lethality in the middle-aged Mexican population is high, and that the population aged 50 to 59 years is particularly vulnerable with a high incidence and lethality, regardless of the presence of risk factors. It has been speculated that this may be the result of the continuous influenza vaccination in the population currently covered, which may cause the shift of the burden of disease to this group [47]. However, at present, there is a lack of serological evidence to prove such a hypothesis. It is worth noting potential barriers to implementation of influenza vaccination that may contribute to the high lethality observed for this population group. Although influenza vaccine uptake by adults in Mexico is reported to be over 95%, mostly due to different immunization promotion strategies that have been implemented over the years, there are still factors that can limit access to vaccination by adults, such as supply chain problems that affect availability of vaccines in clinics, a lack of understanding of the importance of adult vaccination by some sectors of the population, lack of promotion of immunization by healthcare professionals due to insufficient training, and misconceptions, myths and fears related to vaccines [48].

Literature shows that there are important variations in influenza vaccination policies among regions and countries that do not necessarily have a scientific basis [49]. Vaccination against influenza in the elderly (≥60 years of age) is a common practice; nonetheless, there is scarce information regarding the impact of vaccinating younger adult population groups. Reliable data on the impact of influenza, such as those presented here, shed light on the benefits of implementing universal vaccination against influenza in the population aged 50 to 59 years.

A public policy of vaccinating young working adults may have potential benefits that are supported by economic and epidemiologic evidence. A recent study showed a strong inverse relationship between vaccination coverage in nonelderly adults and influenza-related illness in the elderly [50]. Considering that the population aged 50 to 59 years is still economically active, implementing preventive activities in this population to include vaccination alongside screening for chronic diseases may have synergistic effects. We might expect improved vaccination coverage while simultaneously detecting individuals at risk, especially for those with unknown comorbidities. In summary, the results of this analysis support the implementation of a universal influenza vaccination policy in Mexico for the population aged 50 to 59 years old.

Our study has several limitations. The databases used in this study have gaps in duration which may have affected our results. Additionally, there were no influenza vaccine coverage data available for people aged 50 to 59 years with risk factors. As noted in the text, SISVEFLU collects information related to trends in circulating viral strains and the occurrence of severe influenza cases. Therefore, the data from SISVEFLU may have overrepresented the number of severe cases of influenza, thus affecting the lethality rates reported in our study. Most of the monitoring health units included in the SISVEFLU database belong to public health institutions, and there may be bias from not including most private health care facilities; however, it is worth noting that the economic analysis was made using national estimates and costs estimated from the three largest public health providers, which cover >90% of the population in Mexico. Regarding the economic analysis undertaken in this study, out-of-pocket expenses other than the cost of the over-the-counter drugs, amantadine and paracetamol, for patients who did not seek medical care were not considered when making assumptions regarding the economic impact of influenza. This was done to be conservative in estimating the economic benefits of the influenza vaccine; but in doing so, we may have under-estimated such benefits. In line with this, we did not estimate the costs of influenza treatment in patients with comorbidities, since we were analyzing the effects of influenza vaccination to this age group; however, we may have underestimated the costs of influenza management both for the current policy, and after immunization to this group is implemented. We assumed a 100% employment rate in people who acquired influenza, which may overestimate the benefits of influenza immunization in terms of productivity loss. Nonetheless, even if the employment rate was 0%, influenza immunization to the population aged 50 to 59 years without risk factors would result in USD 41.73 million net savings, mostly from direct costs. In Mexico, oseltamivir requires a prescription while amantadine does not. Because of this, we assumed over-the-counter amantadine was used to treat symptoms of influenza by those in Scenario 0, who did not seek medical care. We acknowledge that this assumption may be a limitation of our modelling. Finally, because there are no available data to estimate incidence in Mexico, we used estimated illness rate data from the CDC. The use of surrogate data for indirect standardization is epidemiologically accepted when local data are not available, provided there is reasonable justification. High-quality data regarding the incidence of influenza in the US were available from the CDC. Furthermore, health authorities in both the US and Mexico recognize that infectious disease dynamics, particularly for those transmitted via person-to-person, exhibit similar epidemiological behavior in both countries [51], which may be attributable to the high frequency of migration between the two countries. Retrospective analyses of data from the 2009 influenza pandemic have shown that the identified origins occurred nearly simultaneously in Mexico and the US [52], providing further support for the use of surrogate data. In the last decade, the timing and viral type patterns have been very similar regarding the general epidemiological behavior of infectious diseases [53]. Because the US and Mexico share a border in the same hemisphere and the fact that similar epidemiological behavior of disease is documented in both countries, we believe that our determination that the CDC (US) data are the best available source for influenza incidence estimation in Mexico is supported. Moreover, from the economic point of view, the use of incidence rates from a country where the influenza vaccine is universally applied results in a conservative estimation.

## 5. Conclusions

Our study suggests that influenza in the population aged 50 to 59 years has a high lethality and mortality, similar to population aged ≥60 years who are targeted for vaccination by the current Mexican immunization guidelines. The number of estimated cases in this population during the last decade ranged from 0.3 to 2.9 million cases each year, representing a substantial economic impact for the health care system. In addition, economic analysis showed that vaccination of the population aged 50 to 59 years, who are without risk factors for influenza complications, is cost-saving and a dominant strategy with respect to the current policy. Therefore, expanding the current vaccination schedule to include this population is supported. The national estimates for Mexico as a middle-income country provided from this study will be of great value for health care decision makers in other similar middle-income countries.

## Figures and Tables

**Table 1:** Clinical management by scenarios.

	No Medical Care	Outpatient Only	Hospitalization: Referred from Outpatient Clinic		Hospitalization: Admitted through ER			
Scenario	0	1	2	3	4	5	6	7
Health Outcome	Not Demanding Medical Care	Outpatient Only	Hospitalization, Discharge	Hospital Care, Death	Outpatient	Hospitalization, Non-Severe	Hospitalization, Severe	Hospitalization, Death
Clinical diagnosis								
Outpatient consultations ^1^		1	1	1				
ER consultations ^1^					1	1	1	1
PCR		0.1	0.1	0.1	1	1	1	1
Direct costs								
Outpatient consultations ^1^		1						
Specialist consultations ^1^			2	3	1	2	3	4
Amantadine ^2^	1							
Oseltamivir ^2^		1	1	1	1	1	1	1
Paracetamol ^2^	1	1	1	1	1	1	1	1
Bacteriologic culture ^2^			1	1		1	1	1
Ceftriaxone ^2^			1	1		1	1	1
Hospitalization days			1	8.3	2	8.3	12.45	8.3
Indirect costs								
Medical disability days		3	8		5	15	26	
Years of life lost ^3^				X				X

^1^ Data represent the number of consultations. ^2^ Data represent the number of prescriptions, units purchased (over-the-counter medications), or culture tests ordered. ^3^ Estimation of the indicator (years of life lost) applies only to the populations in the scenarios indicated by X. Abbreviations: ER, emergency room; PCR, polymerase chain reaction.

**Table 2:** Confirmed cases and deaths in the target population ^1^.

	Confirmed Cases									Deaths								
Initial Classification ^2^	ILI			SARI			Total			ILI			SARI			Total		
Season ^3^	Influenza Type			Influenza Type			Influenza Type			Influenza Type			Influenza Type			Influenza Type		
	A	B	Total	A	B	Total	A	B	Total	A	B	Total	A	B	Total	A	B	Total
2009–2010 ^4^	87	1	88	67	0	67	154	1	155	4	0	4	5	0	5	9	0	9
2010–2011	116	5	121	78	1	79	194	6	200	0	0	0	1	0	1	1	0	1
2011–2012	235	3	238	173	1	174	408	4	412	2	0	2	19	0	19	21	0	21
2012–2013	90	22	112	47	18	65	137	40	177	0	0	0	2	1	3	2	1	3
2013–2014	249	12	261	352	10	362	601	22	623	18	0	18	78	1	79	96	1	97
2014–2015	40	33	73	67	30	97	107	63	170	4	0	4	4	0	4	8	0	8
2015–2016	202	82	284	254	44	298	456	126	582	10	1	11	44	2	46	54	3	57
2016–2017	101	60	161	182	41	223	283	101	384	2	1	3	43	1	44	45	2	47
2017–2018	108	25	133	66	17	83	174	42	216	1	1	2	10	4	14	11	5	16
2018–2019 ^5^	10	2	12	2	2	4	12	4	16	1	0	1	0	0	0	1	0	1
Period 2009–2019	1238	245	1483	1288	164	1452	2526	409	2935	42	3	45	206	9	215	248	12	260

^1^ Population aged 50 to 59 years without risk factors for influenza complications. ^2^ Mexico’s current epidemiological surveillance system follows a step-wise process in which health personnel initially classifies cases as either ILI or SARI according to clinical manifestations and, after laboratory test, confirms or discards these cases as influenza. ^3^ A season was defined as beginning in epidemiological week 34 of year 1 (mid-August), and ending in epidemiological week 33 (early August) of year 2. ^4^ The 2009–2010 season includes records starting from November 2009. ^5^ The 2018–2019 season includes only records from August to October 2018. The data used in this table were obtained from SISVEFLU. Abbreviations: ILI, influenza-like illness; SARI, severe acute respiratory infection; SISVEFLU, Mexico’s Influenza Surveillance System.

**Table 3:** Cases, deaths and lethality in the target population ^1^ by season.

Influenza Type	Subtype	Indicator	2009–2010	2010–2011	2011–2012	2012–2013	2013–2014	2014–2015	2015–2016	2016–2017	2017–2018	2018–2019	Period 2009–2019
A	H1N1	Cases	128	11	366	16	509	27	316	184	65	11	1633
		Deaths	8	0	19	0	94	5	50	41	7	1	225
		Lethality (%)	6.25%	0%	5.19%	0%	18.47%	18.52%	15.82%	22.28%	10.77%	9.09%	13.78%
	H3N2	Cases	6	77	5	103	73	77	128	91	103	1	664
		Deaths	0	0	0	2	2	2	2	3	3	0	14
		Lethality (%)	0%	0%	0%	1.94%	2.74%	2.60%	1.56%	3.30%	2.91%	0%	2.11%
	Not subtyped	Cases	20	106	37	18	19	3	12	8	6	0	229
		Deaths	1	1	2	0	0	1	2	1	1	0	9
		Lethality (%)	5.00%	0.94%	5.41%	0%	0%	33.33%	16.67%	12.50%	16.67%	0%	3.93%
	Total A	Cases	154	194	408	137	601	107	456	283	174	12	2526
		Deaths	9	1	21	2	96	8	54	45	11	1	248
		Lethality (%)	5.84%	0.52%	5.15%	1.46%	15.97%	7.48%	11.84%	15.90%	6.32%	8.33%	9.82%
B	Victoria	Cases	0	0	1	2	4	1	4	1	5	0	18
		Deaths	0	0	0	0	0	0	0	0	0	0	0
		Lethality (%)	0%	0%	0%	0%	0%	0%	0%	0%	0%	0%	0%
	Yamagata	Cases	0	0	0	8	4	24	34	34	25	2	131
		Deaths	0	0	0	0	0	0	0	1	3	0	4
		Lethality (%)	0%	0%	0%	0%	0%	0%	0%	2.94%	12.00%	0%	3.05%
	Undetermined lineage	Cases	1	6	3	30	14	38	88	66	12	2	260
		Deaths	0	0	0	1	1	0	3	1	2	0	8
		Lethality (%)	0%	0%	0%	3.33%	7.14%	0%	3.41%	1.52%	16.67%	0%	3.08%
	Total B	Cases	1	6	4	40	22	63	126	101	42	4	409
		Deaths	0	0	0	1	1	0	3	2	5	0	12
		Lethality (%)	0%	0%	0%	2.50	4.55	0%	2.38%	1.98%	11.90%	0%	2.93%
Total influenza		Cases	155	200	412	177	623	170	582	384	216	16	2935
		Deaths	9	1	21	3	97	8	57	47	16	1	260
		Lethality (%)	5.81%	0.50%	5.10%	1.69%	15.57%	4.71%	9.79%	12.24%	7.41%	6.25%	8.86%

The data used in this table were obtained from Mexico’s influenza surveillance system, SISVEFLU. ^1^ Population aged 50 to 59 years without risk factors for influenza complications.

**Table 4:** Hospital discharges with a main diagnosis of influenza (50 to 59 years) (2010–2016).

ICD-10	Main Diagnosis	Discharges	Total Bed-Days	Average Bed-Days
J09X	Influenza due to certain identified influenza virus	162	1851	11.4
J100	Influenza with pneumonia, other influenza virus identified	92	1007	10.9
J101	Influenza with other respiratory manifestations, other influenza virus identified	56	347	6.2
J108	Influenza with other manifestations, other influenza virus identified	15	62	4.1
J110	Influenza with pneumonia, virus not identified	307	2923	9.5
J111	Influenza with other respiratory manifestations, virus not identified	394	2431	6.2
J118	Influenza with other manifestations, virus not identified	39	210	5.4
Total		1065	8831	8.3

The data used in this table were obtained from the SAEH [24]. Abbreviation: ICD-10, International Statistical Classification of Diseases and Related Health Problems, 10th revision; SAEH, Automated Hospital Discharge System.

**Table 5:** Mortality by age group and season (per 100,000 inhabitants).

Age Group	Season ^1^					Average Mortality
	2010–2011	2011–2012	2012–2013	2013–2014	2014–2015	
<1	0.8	1.3	1.0	1.2	0.4	0.9
1–4	0.1	0.1	0.4	0.2	0.1	0.2
5–11	0.0	0.0	0.0	0.1	0.0	0.0
12–17	0.1	0.0	0.0	0.1	0.0	0.0
18–49	0.1	0.2	0.0	0.7	0.0	0.2
50–59	0.8	0.9	0.0	2.0	0.1	0.8
≥60	1.3	1.0	0.1	2.0	0.3	0.9
Total	0.2	0.3	0.1	0.8	0.1	0.3

**^1^** The 2009–2010 and 2015–2016 seasons were not analyzed, since the data were not complete. The data used in this table were obtained from SEED [21] and CONAPO [24]. Abbreviations: CONAPO, National Population Council; SEED, National Death Epidemiological and Statistics Subsystem.

**Table 6:** Estimated influenza-related events averted by immunizing the target population ^1^.

Outcomes	No Influenza Immunization	Influenza Immunization	Influenza-Related Events Averted
Influenza cases	797,918	598,418	199,500
Outpatient consultations	268,004	200,996	67,008
Specialty consultations	215,136	161,347	53,790
Emergency room consultations	132,082	99,058	33,024
Hospitalizations	132,349	99,258	33,091

^1^ Population aged 50 to 59 years without risk factors for influenza complications.

**Table 7:** Total economic costs of influenza treatment of target population ^1^ without influenza immunization (million US dollars).

Scenario	0	1	2	3	4	5	6	7	Cost
Direct costs									
Laboratory diagnosis		1.55	0.01	0.0000	8.27	4.87	2.78	0.01	17.48
Medical consultations		6.37	0.03	0.0001	4.55	3.90	3.05	0.01	17.90
Drugs	1.39	0.82	0.00	0.0000	0.44	0.26	0.15	0.00	3.06
Hospitalizations				0.0018	32.32	72.33	61.80	0.09	166.61
Total direct costs	1.39	8.74	0.09	0.00	45.57	81.37	67.78	0.10	205.04
Indirect costs									
Productivity loss	0.00	6.78	0.04		6.04	9.77	9.65		32.27
Premature death				0.08				3.88	3.96
Total indirect costs	0.00	6.78	0.04	0.08	6.04	9.77	9.65	3.88	36.23
Total costs of influenza	1.39	15.52	0.13	0.08	51.61	91.14	77.42	3.99	241.27

^1^ Population aged 50 to 59 years without risk factors for influenza complications.

**Table 8:** Costs of vaccination of the target population ^1^ (US dollars).

Population to Vaccinate	Cost (US Dollars)
Total population 50–59 years	6,444,501
At 50% vaccination coverage	
Vaccinated population	3,222,250
Cost of immunization	
Cost per dose	3.002
Application of vaccine	0.236
Storage and transportation	0.028
Unit cost per person immunized	3.267
Total cost of immunization	
Population 50–59 years	10,525,569.95

^1^ Population aged 50 to 59 years without risk factors for influenza complications.

**Table 9:** Costs averted (million US dollars).

	No Influenza Immunization	Influenza Immunization	Net Costs
Direct costs			
Laboratory diagnosis	17.48	13.11	−4.37
Medical consultations	17.90	13.42	−4.48
Drugs	3.06	2.30	−0.77
Hospitalizations	166.61	124.95	−41.66
Vaccination population aged 50–59 years		10.53	10.53
Net direct costs (Third party payer perspective)	205.04	164.30	−40.74
Indirect costs			
Productivity loss	32.27	24.20	−8.07
Premature death	3.96	2.97	−0.99
Net indirect costs	36.23	27.17	−9.06
Net total costs of influenza (Societal perspective)	241.27	191.47	−49.80

**Table 10:** Estimated influenza cases averted by immunizing the target population ^1^: Sensitivity analysis.

Scenario	Base Case	Conservative	Optimistic
0–Not demanding medical care	132,939	30,314	253,110
1–Outpatient only	33,471	7632	63,727
2–Hospitalization, discharge	67	15	127
4–Outpatient	17,885	4078	34,053
5–Hospitalization, non-severe	9645	2199	18,363
6–Hospitalization, severe	5494	1253	10,460
Total	199,500	45,493	379,840

^1^ Population aged 50 to 59 years without risk factors for influenza complications.

**Table 11:** Estimated influenza-related events avoided by immunizing the population aged 50 to 59 years: Sensitivity analysis.

Events Avoided	Base Case	Conservative	Optimistic
Vaccination coverage	50%	30%	70%
Vaccine effectiveness	50%	19%	68%
Influenza cases	199,500	45,493	379,840
Outpatient consultations	67,008	15,280	127,580
Specialty consultations	53,790	12,266	102,414
Emergency room consultations	33,024	7531	62,876
Hospitalizations	33,091	7546	63,003
Deaths	12.1	2.8	23.0

**Table 12:** Total economic benefits of influenza immunization to the target population ^1^: Sensitivity analysis.

Influenza-Associated Costs Averted	Base Case	Conservative	Optimistic
Vaccination coverage	50%	30%	70%
Vaccine effectiveness	50%	19%	68%
Direct Costs			
Laboratory diagnosis	−4.37%	−1.00%	−8.3%
Medical consultations	−4.48%	−1.02%	−8.5%
Drugs	−0.77%	−0.17%	−1.5%
Hospitalizations	−41.66%	−9.50%	−79.3%
Influenza immunization 50–59 years	+10.53%	+6.32%	+14.7%
Total direct costs (Third-party payer perspective)	−40.74%	−5.38%	−82.9%
Indirect costs			
Productivity loss	−8.07%	−1.84%	−15.4%
Premature death	−0.99%	−0.23%	−1.9%
Total indirect costs	−9.06%	−2.07%	−17.2%
Total costs of influenza	−49.80%	−7.44%	−100.1%

^1^ Population aged 50 to 59 years without risk factors for influenza complications.

## Data Availability

All of the datasets generated and/or analyzed during the current study are available in the *Bases de datos sobre defunciones* (death databases) repository, http://www.dgis.salud.gob.mx/contenidos/basesdedatos/std_defunciones_gobmx.html (accessed on 20 February 2021), and the *Egresos hospitalarios Secretaría de Salud* (hospital discharges, Ministry of Health) repository, www.dgis.salud.gob.mx/contenidos/basesdedatos/da_egresoshosp_gobmx.html (accessed on 20 February 2021), with the exception of data from influenza cases obtained from SISVEFLU, which were analyzed with permission from Mexico’s General Directorate of Epidemiology (Ministry of Health) and are not publicly available in compliance with Mexico’s Personal Data Protection Law (http://www.diputados.gob.mx/LeyesBiblio/pdf/LFPDPPP.pdf (accessed on 20 February 2021)).

## Supplementary Materials

The following are available online at https://www.mdpi.com/2076-393X/9/3/188/s1, Text S1. Methodology for calculation of national estimates, Table S1.1. Influenza cases per 100,000 people in the United States by age group, Table S1.2. Estimation of influenza cases in Mexico, Text S2. Methodology for the estimation of the different scenarios, Table S2.1. Confirmed cases of influenza per season and institution, population aged 50 to 59 years, Table S2.2. Classification of cases in each scenario according to health outcome, Table S2.3. Health outcomes and classification by scenario. Results from SISVEFLU, Table S2.4. Distribution of cases per season and scenario, Table S3.1. Prevalence of the population aged 50 to 59 years with risk factors for influenza complications, Table S3.2. Cases, deaths and lethality in the population aged 50 to 59 years (2009–2018), Table S4.1. Unit costs (Mexican pesos) using 2018 prices from the three largest public health care providers in Mexico, Table S4.2. Unit cost (Mexican pesos) by public health provider and season, Table S4.3. Affiliated population by public health provider and season, Table S4.4. Average weighted cost (Mexican pesos) by season, Table S5. Effectiveness of influenza vaccination in the Northern Hemisphere, Table S6.1. Estimated influenza cases averted by immunizing the target population, Table S6.2. Deaths averted by immunizing the target population, Table S6.3. Total economic costs of influenza treatment if the target population is immunized, Text S3. Cases, deaths, and lethality by viral type (2009–2018), Table S7.1. Confirmed influenza cases by age group and season, Table S7.2. Cases, deaths, and lethality by viral type (2009–2018), Table S7.3. Estimated influenza cases by age group and season, Table S7.4. Lethality by age group and season, Table S7.5. Years of life lost per age group by season, Figure S1. Projection of population aged 50–59 years and total population in Mexico (2010–2030).
