# Supplementary material for: Public Health and Economic Benefits of Influenza Vaccination of the Population Aged 50 to 59 Years without Risk Factors for Influenza Complications in Mexico: A Cross-Sectional Epidemiological Study
Source: Vaccines (Basel). 2021 Feb 24;9(3):188. doi: 10.3390/vaccines9030188 (PMC7995959; doi:10.3390/vaccines9030188)
Supplement: Supplementary file 1 [file vaccines-09-00188-s001.zip › vaccines-1097272-supplementary.pdf]

vaccines

MDPI

Supplementary File

Public health and economic benefits of influenza vaccination of the population aged 50 to 59 years without risk factors for influenza complications in Mexico: a cross-sectional epidemiological study

Miguel Betancourt-Cravioto[1], Jorge Abelardo Falcón-Lezama[1], Rodrigo Saucedo-Martínez[1], Myrna María Alfaro-Cortes[1], Roberto Tapia-Conyer[1,2]

[1]Sociedad Mexicana de Salud Pública, Herschel 109, Anzures, Miguel Hidalgo, 11590 Mexico City, Mexico

[2]Universidad Nacional Autónoma de México, Av. Universidad 3000, Circuito Escolar CU, Edificio B 1er Piso, Coyoacan, 04510 Mexico City, Mexico

Corresponding author:

Roberto Tapia-Conyer

Lago Zurich 245, Presa Falcon Floor 20, Col. Ampliacion Granada, Miguel Hidalgo, 11529 Mexico City, Mexico

Tel: +52 (55) 5339 1796

Email: tapiaconyer@yahoo.com.mx

vaccines MDPI

Supplement 1

Text S1. Methodology for calculation of national estimates

Main characteristics of SISVEFLU

The SISVEFLU (Influenza Epidemiologic Surveillance System) was developed by the General Directorate of Epidemiology of Mexico's Ministry of Health with the objective of collecting epidemiologic data about trends in circulating strains of influenza virus, as well as other causative agents of severe acute respiratory infection (SARI) [27].

The SISVEFLU system operates through monitoring health facilities that were selected for convenience. Because of this, the collected data are not considered to be representative of the population. Facilities, both primary health care clinics and hospitals, must comply with at least one of the following criteria:

- They must be willing to be part of the SISVEFLU.
- They serve a relatively large population.
- They have adequate medical staff sufficiently specialized to diagnose, clinically manage, and report influenza cases.
- They have, or have access to, a high-quality diagnostic laboratory.

Burden of disease estimation

SISVEFLU's ultimate goal is to identify and confirm influenza cases and trends, as well as deaths, attributed to both influenza and other SARIs. According to the epidemiological surveillance

guidelines, sample collection for confirmation is considered in only 10% of cases in primary health care facilities, whereas in secondary and tertiary health care facilities, samples must be submitted for confirmation in 100% of cases. In light of this, reporting of cases from primary care clinics may be underestimated compared with that reported from hospitals. Unlike primary care clinics, hospitals do not have a defined population because they receive cases from different geographical areas. Thus, information from these facilities cannot be extrapolated. According to the World Health Organization's methodology to estimate burden of disease due to influenza, data extrapolation from the SISVEFLU is limited because it is not representative of the population. Moreover, as noted from the data collected and reported in the article, most type B influenza cases did not have lineage analysis; therefore, data cannot be extrapolated for either the Victoria or Yamagata lineages.

As an alternative, we used the standardized influenza incidence data for the United States, estimated by the Centers for Disease Control and Prevention (CDC) for the same seasons (**Table S1.1**). We chose the United States because it is in the Northern Hemisphere and shares viral circulation with Mexico. The use of surrogate data for indirect standardization is epidemiologically accepted when local data are not available, provided there is reasonable justification. High-quality data regarding the incidence of influenza in the US were available from the CDC. Furthermore, health authorities in both the US and Mexico recognize that infectious disease dynamics, particularly for those transmitted via person-to-person, exhibit similar epidemiological behavior in both countries [51], which may be attributable to the high frequency of migration between the two countries. Ret-

rospective analyses of data from the 2009 influenza pandemic have shown that the identified origins occurred nearly simultaneously in Mexico and the US [52], providing further support for the use of surrogate data. In the last decade, the timing and viral type patterns have been very similar regarding the general epidemiological behavior of infectious diseases [53]. The US and Mexico share a border in the same hemisphere and the fact that similar epidemiological behavior of disease is documented in both countries supports the use of CDC (US) data as the best available source for influenza incidence estimation in Mexico.

Table S1.2 shows national estimates obtained by indirectly standardizing CDC estimates to the population of Mexico, taking the same age groups into consideration. For this exercise, data on Mexican population projections for the study period were obtained from the National Population Council database at the beginning of each year [26]. Data regarding state estimates are available upon request.

Table S1.1. Influenza cases per 100,000 people in the United States by age group [33]

Season	0–4 years	5–17 years	18–49 years	50–64 years	≥65 years
**2010–2011**	13,743.20	8216.60	5468.10	8240.50	4521.10
**2011–2012**	4697.10	3711.80	2564.20	3181.40	2333.60
**2012–2013**	17,820.50	12,419.20	8383.60	12,852.10	9712.10
**2013–2014**	12,711.70	7416.10	9589.60	13,712.90	3819.30
**2014–2015**	16,135.90	11,895.00	6310.30	11,626.40	10,120.20
**2015–2016**	11,498.70	8094.10	7390.70	11,200.80	3460.60
**2016–2017**	11,478.30	11,083.30	6227.90	13,654.80	9013.80
**2017–2018**	19,983.70	13,985.60	10,469.70	24,588.10	14,371.40
**2018–2019**[1]	13,508.64	9602.71	7050.51	12,382.13	7169.01

[1] Incidence for season 2018–2019 was estimated for each age group as the average of seasons 2010–2011 to 2017–2018.

Table S1.2. Estimation of influenza cases in Mexico

Season	Population	Estimated Cases	Cases per 100,000
**2010–2011**	114,551,762	8,275,119	7223.91
**2011–2012**	116,179,714	3,616,140	3112.54
**2012–2013**	117,668,241	12,803,672	10,881.16
**2013–2014**	119,216,240	11,281,198	9462.80
**2014–2015**	120,653,293	11,413,739	9459.95
**2015–2016**	122,038,924	9,928,898	8135.85
**2016–2017**	123,388,022	11,074,556	8975.39
**2017–2018**	124,692,044	17,749,078	14,234.33
**2018–2019**	125,960,168	11,223,972	8910.73
**TOTAL**		**97,366,372**	

Supplement 2

Text S2. Methodology for the estimation of the different scenarios

Number of cases of influenza registered in SISVEFLU

The SISVEFLU (Influenza Epidemiologic Surveillance System) was developed by the General Directorate of Epidemiology of Mexico's Ministry of Health with the objective of collecting epidemiologic data about trends in circulating strains of influenza virus, as well as other causative agents of severe acute respiratory infection (SARI) [27].

From seasons 2009–2010 to 2017–2018, health monitoring units registered 50,900 confirmed cases of influenza, of which 5725 were of people aged 50 to 59 years. These cases were distributed between the different institutions of Mexico's Health System as shown in **Table S2.1**.

Estimated cases were then allocated into eight different scenarios, considering 1) the probability of not demanding medical care (scenario 0), as reported by Molinari et al. for different age groups [34], 2) where cases were diagnosed according to the SISVEFLU database (outpatient clinic or hospital), and 3) likelihood of occurrence of health outcome (ambulatory discharge, hospitalization and subsequent discharge, or death).

Regarding the decision analytics used for the present study, we followed the method of Molinari et al. for cases from scenario 0 (symptomatic individuals who do not seek medical care) and considered the likelihood (0.6664) of seeking medical care for patients with low risk [34]. For estimation of cases for scenarios either from ambulatory or inpatient care that do not end in death (1, 2, 4, 5, and 6), the likelihood of each scenario was calculated considering data registered from health monitoring units at SISVEFLU. Within this database, there is a variable named "Evolution" where health professionals from monitoring units register, for each case, the code that corresponds to the health outcome of that case. **Table S2.2** shows the different codes that were found in the database, and the scenario to which they were allocated. For scenarios either from ambulatory or inpatient care that resulted in death (3 and 7), the number of deaths was obtained directly from SISVEFLU. Hence, following this classification, all 5725 cases were allocated to a single scenario, and based on the date of each case, they were allocated to one season from 2009–2010 to 2017–2018. Based on that, the likelihood of occurrence of each scenario for a typical influenza season was estimated, and these likelihoods were used to project national estimates of influenza. **Table S2.3** shows classification of cases for each of the SISVEFLU codes previously described. Cases were then allocated to its corresponding season based on date of occurrence (**Table S2.4**).

Table S2.1. Confirmed cases of influenza per season and institution, population aged 50 to 59 years

**Institution**	**2009–2010**	**2010–2011**	**2011–2012**	**2012–2013**	**2013–2014**	**2014–2015**	**2015–2016**	**2016–2017**	**2017–2018**	**2018–2019**	**TOTAL PER INSTITUTION**
SSA	128	151	426	176	582	153	568	322	185	20	**2711**
IMSS	93	127	212	112	461	142	456	380	188	30	**2201**
ISSSTE	18	6	63	14	158	21	125	104	48	5	**562**
IMSS-OPORTUNIDADES	0	1	3	2	7	1	9	2	3	1	**29**
PEMEX	3	5	1	0	4	2	2	7	3	0	**27**
SEDENA	0	0	1	0	0	0	0	0	0	0	**1**
SEMAR	0	0	0	0	0	0	0	0	0	0	**0**
DIF	0	0	0	0	0	0	0	0	0	0	**0**
STATE	0	0	9	1	5	5	23	8	4	1	**56**
UNIVERSITY	5	0	1	0	3	4	9	9	2	0	**33**
PRIVATE	9	15	6	2	8	6	17	7	6	1	**77**
NOT IDENTIFIED	28	0	0	0	0	0	0	0	0	0	**28**
**TOTAL**	**284**	**305**	**722**	**307**	**1228**	**334**	**1209**	**839**	**439**	**58**	**5725**

Source: SISVEFLU

Abbreviations: SSA, Secretaría de Salubridad y Asistencia (the Federal Secretariat of Health), IMSS, Instituto Mexicano del Seguro Social (Mexican Social Security Institute); ISSSTE, Instituto de Seguridad y Servicios Sociales para los Trabajadores del Estado (Institute for Social Security and Services for State Workers); IMSS-OPORTUNIDADES, IMSS-Opportunities; PEMEX, Petroleos Mexicanos (Mexican Petroleum); SEDENA, Secretaria de Defensa Nacional (Secretariat of National Defense); DIF, Sistema Nacional para el Desarrollo Integral de la Familia (National System for Integral Family Development).

Table S2.2. Classification of cases in each scenario according to health outcome

**SISVEFLU Code**	**Description**	**Scenario**
**Patients who seek ambulatory care**		
DISCHARGE	Patient who sought ambulatory care and was discharged. It is not clear if the patient continues pharmacological treatment after discharge; nonetheless the costs of drug therapy are already executed.	1
UNDER TREATMENT	Patient who sought ambulatory care, was discharged, and continued treatment.	1

SISVEFLU Code	Description	Scenario
HOUSEHOLD FOLLOW-UP	Patient who sought ambulatory care, was discharged, and continued treatment with household visits.	1
FINALISED TREATMENT	Patient who sought ambulatory care, was discharged, and finalized treatment.	1
REFERRAL	Patient who sought ambulatory care and was referred to a hospital for follow-up	2
DEATH	Patient who sought ambulatory care and resulted in death.	3
NOT APPLICABLE	Patient who sought ambulatory care and whose health outcome is unknown. Given that health monitoring units follow a comprehensive protocol to register the final outcome of each case, it is inferred that these cases did not have any complications, and the outcome was discharge.	1
**Patients who seek inpatient care**		
UNDER TREATMENT	Patient who sought inpatient care, was discharged, and continued treatment in an ambulatory unit. In this case, we assume that patients only entered the emergency room and were not hospitalized during their stay.	1
HOUSEHOLD FOLLOW-UP	Patient who sought inpatient care, was discharged, and was monitored through household visits from primary care or ambulatory units.	1
DISCHARGE CURATION	Patient who sought inpatient care, began treatment, and was discharged for monitoring through a primary care or ambulatory unit.	4
DISCHARGE IMPROVEMENT		
DISCHARGE TRANSFER		
VOLUNTARY DISCHARGE		
NON-SEVERE CASE	Patient who sought inpatient care, was hospitalized, began treatment, and was classified as a non-severe case. Outcome was complete recovery and discharge.	5
SEVERE CASE	Patient who sought inpatient care, was hospitalized, began treatment, and was classified as a severe case. Outcome was complete recovery and discharge.	6
REFERRAL	Patient who sought inpatient care, was hospitalized, began treatment, and due to the severity of the case, was referred to a tertiary hospital. Outcome was complete recovery and discharge.	6
DEATH	Patient who sought inpatient care, was hospitalized, began treatment, and did not respond to it. Outcome was death.	7

Source: Author’s elaboration using SISVEFLU codes

Table S2.3. Health outcomes and classification by scenario. Results from SISVEFLU

Health outcome	Number of cases	Total number of cases	Scenario
**Ambulatory care**			
UNDER TREATMENT	1546	2309	1
HOUSEHOLD FOLLOW-UP	33		
FINALISED TREATMENT	121		
NOT APPLICABLE	609		
REFERRAL	5	5	2

Health outcome	Number of cases	Total number of cases	Scenario
DEATH	16	16	3
**Total ambulatory care**	**2330**	**40.7%**	
**Inpatient care**			
DISCHARGE CURATION	31	1492	4
DISCHARGE IMPROVEMENT	23		
DISCHARGE TRANSFER	1414		
VOLUNTARY DISCHARGE	24		
NON-SEVERE CASE	690	690	5
SEVERE CASE	384	394	6
REFERRAL	10		
DEATH	819	819	7
**Total inpatient care**	**3395**	**59.3%**	
**Total number of cases**		**5725**	

Source: Author's elaboration using SISVEFLU codes.

Table S2.4. Distribution of cases per season and scenario

Scenario	2009–2010	2010–2011	2011–2012	2012–2013	2013–2014	2014–2015	2015–2016	2016–2017	2017–2018	2018–2019	TOTAL	
1	108	215	368	205	399	150	425	273	150	16	**2309**	**40.3%**
2	0	1	3	0	1	0	0	0	0	0	**5**	**0.1%**
3	0	0	0	0	6	2	6	2	0	0	**16**	**0.3%**
4	52	28	116	51	292	64	409	321	147	12	**1492**	**26.1%**
5	48	37	109	22	190	80	95	58	42	9	**690**	**12.1%**
6	41	17	50	18	78	16	67	51	42	14	**394**	**6.9%**
7	35	7	76	11	262	22	207	134	58	7	**819**	**14.3%**
TOTAL	284	305	722	307	1228	334	1209	839	439	58	5725	**100.0%**

Source: Author's elaboration using SISVEFLU codes

Supplement 3

Table S3.1. Prevalence of the population aged 50 to 59 years with risk factors for influenza complications

Condition	Prevalence in the population aged 50–59 years	Reference
**Respiratory conditions**		

COPD[1]	2.60	[35]
Asthma	2.28	[35]
**Cardiovascular conditions**		
Stroke[2]	0.90	[35]
Angina	2.00	[35]
Myocardial infarction[3]	1.70	[35]
Heart failure	1.60	[35]
**Metabolic conditions**		
Diabetes[4]	10.55	[35]
Morbid obesity	6.68	[35]
Uncontrolled hypertension	12.42	[35]
**Immunity disorders & cancer**		
AIDS/HIV	0.20	[35]
Cancer	0.30	[35]
Chronic Kidney Disease[5]	6.08	[35]
Pregnancy	0.00	[35]
Asplenia	0.01	[35]
Sickle cell disease	0.01	[35]
	**47.33**	

[1] Corrected for double counting patients with asthma, assuming 40% of COPD patients also have asthma based on van der Molen et al. [36].

[2] Corrected for double counting patients with heart failure, assuming 16.5% of stroke survivors also have heart failure based on Balzi et al. [37].

[3] Corrected for double counting patients with angina, heart failure and stroke; assuming 26% of myocardial infarction survivors also have angina [38], 6.5% also have heart failure [38], and 8% have a history of stroke [37].

[4] Corrected for double counting patients with obesity, assuming 41.9% of diabetes patients are also obese based on Nguyen et al. [39].

[5] Corrected for double counting patients with hypertension, assuming 40.92% of CKD patients have uncontrolled hypertension; corrected for double counting patients with diabetes, assuming 17% of CKD patients are also diabetic. Based on Fraser et al. [40].

Abbreviations: AIDS, acquired immune deficiency syndrome; CKD, chronic kidney disease; COPD, chronic obstructive pulmonary disease; HIV, human immunodeficiency virus.

Table S3.2. Cases, deaths and lethality in the population aged 50 to 59 years (2009–2018)

Risk factors[1]	Cases	%	Deaths	%	Lethality %
**Not known or absent**	2935	51.27	260	31.21	8.86
**Previously known**	2790	48.73	573	68.79	20.54
**Total**	**5725**	**100**	**833**	**100**	**14.55**

Source: SISVEFLU

[1] Diabetes, COPD, asthma, immunosuppression, HIV infection /AIDS, cardiac disease, obesity, chronic kidney disease, and pregnancy.

Abbreviations: AIDS, acquired immunodeficiency syndrome; COPD, chronic obstructive pulmonary disease; HIV, human immunodeficiency virus; SISVEFLU, Mexico's Influenza Surveillance System

Supplement 4

Table S4.1. Unit costs (Mexican pesos) using 2018 prices from the three largest public health care providers in Mexico

**Item**	**Cost ISSSTE**	**Cost IMSS**	**Cost SSA**
Outpatient consultation	370.48	733.00	98.80
Specialist consultation	811.53	1160.00	98.80
Emergency room care	811.53	562.00	305.21
PCR	2380.47	1932.43	2380.47
Bacteriologic culture	201.34	201.34	201.34
Amantadine	45.00	45.00	45.00
Oseltamivir	110.87	110.87	110.87
Paracetamol	9.30	3.12	3.12
Ceftriaxone	11.87	11.89	10.67
Hospitalization bed-day	2064.12	7757.00	305.21

Abbreviations: IMSS, Instituto Mexicano del Seguro Social (Mexican Social Security Institute); ISSSTE, Instituto de Seguridad y Servicios Sociales para los Trabajadores del Estado (Institute for Social Security and Services for State Workers); SSA, Secretaría de Salubridad y Asistencia (the Federal Secretariat of Health); PCR, polymerase chain reaction.

Data sources for each unit cost are the following:

ISSSTE

- Outpatient consultation, specialist consultation, emergency room (ER) care, and hospitalization bed-day: publicly available data from Mexico's Ministry of Health (Secretaría de Salud, http://www.salud.gob.mx/unidades/cdi/documentos/DOCSAL7417.pdf., assessed 2 January 2020)
- Amantadine: 2018 drugstore price, sold as an over-the-counter drug; it does not require a prescription and therefore can be purchased directly by the patient (San Pablo Farmacia, https://www.farmaciasanpablo.com.mx/medicamentos/genericos/a---b---c---d/clorhidrato-de-amantadina-50-mg-clorfenamina-3-mg-paracetamol-300-mg/p/000000000060160010., accessed on 5 January 2020).
- Oseltamivir: 2018 price through consolidated government purchase.
- Paracetamol and Ceftriaxone: ISSSTE's 2019 Annual Acquisitions Program (ISSSTE, http://www.issste.gob.mx/images/downloads/instituto/administracion/paaas_2019.pdf., accessed on 5 January 2020). For paracetamol, the code is 25301688; for ceftriaxone, the code is 25300461.
- PCR and throat swab: 2018 price list InDRE.

IMSS

- Outpatient consultation, specialist consultation, ER care, and hospitalization bed-day: Mexico's Official Federal Gazette (SEGOB, http://www.issste.gob.mx/images/downloads/instituto/administracion/paaas_2019.pdf., accessed on 5 January 2020).
- Amantadine: 2018 drugstore public price (ISSSTE, http://www.issste.gob.mx/images/downloads/instituto/administracion/paaas_2019.pdf., accessed on 5 January 2020).
- Oseltamivir: 2018 price through consolidated government purchase.
- Paracetamol: IMSS Acquisitions Portal (Instituto Mexicano del Seguro Social, http://compras.imss.gob.mx/?P=imsscomprofich&f=22409921&pr=., accessed on 5 January 2020).
- Ceftriaxone: IMSS Acquisitions Portal (Instituto Mexicano del Seguro Social., http://compras.imss.gob.mx/?P=imsscomprofich&f=22409744&pr=., accessed on 5 January 2020).
- PCR and throat swab: 2018 price list InDRE.

SSA

- Outpatient consultation, specialist consultation, ER care, and hospitalization bed-day: publicly available data from Mexico's Ministry of Health (Secretaría de Salud, http://www.salud.gob.mx/unidades/cdi/documentos/DOCSAL7417.pdf., assessed 2 January 2020; and SEGOB, http://www.issste.gob.mx/images/downloads/instituto/administracion/paaas_2019.pdf., accessed on 5 January 2020)
- Amantadine: 2018 drugstore public price (ISSSTE, http://www.issste.gob.mx/images/downloads/instituto/administracion/paaas_2019.pdf., accessed on 5 January 2020).
- Oseltamivir: 2018 price through consolidated government purchase.
- Paracetamol: IMSS Acquisitions Portal (Instituto Mexicano del Seguro Social, http://compras.imss.gob.mx/?P=imsscomprofich&f=22409921&pr=., accessed on 5 January 2020).
- Ceftriaxone: IMSS Acquisitions Portal (Instituto Mexicano del Seguro Social., http://compras.imss.gob.mx/?P=imsscomprofich&f=22409744&pr=., accessed on 5 January 2020).
- PCR and throat swab: 2018 price list InDRE.

Table S4.2. Unit cost (Mexican pesos) by public health provider and season

Unit cost	Season								
	2009–2010	2010–2011	2011–2012	2012–2013	2013–2014	2014–2015	2015–2016	2016–2017	2017–2018
**IMSS**									
Outpatient consultation	536.69	554.98	577.80	599.79	623.90	640.87	658.95	698.77	733.00
Specialist consultation	849.34	878.28	914.39	949.20	987.34	1014.20	1042.82	1105.82	1160.00
ER care	411.49	425.51	443.01	459.87	478.35	491.36	505.23	535.75	562.00
Hospitalization bed-day	5679.59	5873.12	6114.59	6347.34	6602.41	6782.04	6973.41	7394.71	7757.00
Amantadine	32.95	34.07	35.47	36.82	38.30	39.34	40.45	42.90	45.00
Oseltamivir	81.18	83.94	87.40	90.72	94.37	96.94	99.67	105.69	110.87
Paracetamol	2.28	2.36	2.46	2.55	2.66	2.73	2.80	2.97	3.12
Ceftriaxone	8.71	9.00	9.37	9.73	10.12	10.40	10.69	11.33	11.89
PCR	1414.90	1463.12	1523.27	1581.25	1644.80	1689.55	1737.22	1842.17	1932.43
Bacteriologic culture	147.42	152.44	158.71	164.75	171.37	176.03	181.00	191.94	201.34
**ISSSTE**									
Outpatient consultation	271.26	280.51	292.04	303.16	315.34	323.92	333.06	353.18	370.48
Specialist consultation	594.20	614.44	639.71	664.06	690.74	709.53	729.56	773.63	811.53
ER care	594.20	614.44	639.71	664.06	690.74	709.53	729.56	773.63	811.53
Hospitalization bed-day	1511.33	1562.82	1627.08	1689.01	1756.89	1804.68	1855.61	1967.71	2064.12
Amantadine	32.95	34.07	35.47	36.82	38.30	39.34	40.45	42.90	45.00
Oseltamivir	81.18	83.94	87.40	90.72	94.37	96.94	99.67	105.69	110.87
Paracetamol	6.81	7.04	7.33	7.61	7.92	8.13	8.36	8.87	9.30
Ceftriaxone	8.69	8.99	9.36	9.71	10.10	10.38	10.67	11.32	11.87
PCR	1742.95	1802.34	1876.45	1947.87	2026.15	2081.27	2140.00	2269.29	2380.47
Bacteriologic culture	147.42	152.44	158.71	164.75	171.37	176.03	181.00	191.94	201.34
**SSA**									
Outpatient consultation	72.34	74.80	77.88	80.84	84.09	86.38	88.82	94.18	98.80
Specialist consultation	72.34	74.80	77.88	80.84	84.09	86.38	88.82	94.18	98.80
ER care	223.47	231.08	240.59	249.74	259.78	266.85	274.38	290.95	305.21
Hospitalization bed-day	223.47	231.08	240.59	249.74	259.78	266.85	274.38	290.95	305.21
Amantadine	32.95	34.07	35.47	36.82	38.30	39.34	40.45	42.90	45.00
Oseltamivir	81.18	83.94	87.40	90.72	94.37	96.94	99.67	105.69	110.87
Paracetamol	2.28	2.36	2.46	2.55	2.66	2.73	2.80	2.97	3.12
Ceftriaxone	7.81	8.08	8.41	8.73	9.08	9.33	9.59	10.17	10.67
PCR	1742.95	1802.34	1876.45	1947.87	2026.15	2081.27	2140.00	2269.29	2380.47
Bacteriologic culture	147.42	152.44	158.71	164.75	171.37	176.03	181.00	191.94	201.34

Data adjusted using Mexico's National Consumer Price Index (INPC).
Abbreviations: IMSS, Instituto Mexicano del Seguro Social (Mexican Social Security Institute); ISSSTE, Instituto de Seguridad y Servicios Sociales para los Trabajadores del Estado (Institute for Social Security and Services for State Workers); ER, emergency room; PCR, polymerase chain reaction.

Table S4.3. Affiliated population by public health provider and season

Season	IMSS[1]	ISSSTE[2]	SSA[3]

2009–2010	50,722,198	11,791,419	37,325,834
2010–2011	53,608,241	12,100,042	47,671,017
2011–2012	56,191,147	12,328,170	52,365,663
2012–2013	58,493,930	12,540,089	54,273,005
2013–2014	59,499,554	12,717,193	56,469,000
2014–2015	60,676,058	12,888,774	57,202,811
2015–2016	62,672,649	13,043,445	56,014,787
2016–2017	64,783,558	13,184,870	54,214,570
2017–2018	67,122,622	13,301,986	53,502,114

[1] IMSS: IMSS. Memoria Estadística 2018. [2018 Statistical Report] Available online: http://www.imss.gob.mx/conoce-al-imss/memoria-estadistica-2018

[2] ISSSTE: ISSSTE. Anuario Estadística 2018 [2018 Annual Statistical Report]. Available online: http://www.issste.gob.mx/datosabiertos/anuarios/anuarios2018.html#cap1

[3] Seguro Popular. Seguro Popular. Beneficiarios de Protección Social en Salud de Seguro Popular. [Popular insurance. Beneficiaries of Social Protection in Popular Health Insurance.] Available online: https://datos.gob.mx/busca/dataset/beneficiarios-de-proteccion-social-en-salud-de-seguro-popular

Abbreviations: IMSS, Instituto Mexicano del Seguro Social (Mexican Social Security Institute); ISSSTE, Instituto de Seguridad y Servicios Sociales para los Trabajadores del Estado (Institute for Social Security and Services for State Workers); SSA, Secretaría de Salubridad y Asistencia (the Federal Secretariat of Health).

Table S4.4. Average weighted cost (Mexican pesos) by season

Unit cost	Season								
	2009–2010	2010–2011	2011–2012	2012–2013	2013–2014	2014–2015	2015–2016	2016–2017	2017–2018
Outpatient consultation	331.74	323.79	332.10	345.34	356.53	367.07	384.25	416.33	443.64
Specialist consultation	528.72	512.30	524.01	544.56	561.67	578.31	606.14	657.76	701.45
ER care	362.78	363.93	375.38	389.29	403.43	414.65	429.28	459.08	484.20
Hospitalization bed-day	3147.49	3040.89	3112.41	3240.17	3340.33	3441.46	3618.09	3939.79	4214.67
Amantadine	32.95	34.07	35.47	36.82	38.30	39.34	40.45	42.90	45.00
Oseltamivir	81.18	83.94	87.40	90.72	94.37	96.94	99.67	105.69	110.87
Paracetamol	2.82	2.86	2.96	3.06	3.18	3.26	3.35	3.56	3.73
Ceftriaxone	8.37	8.61	8.95	9.30	9.66	9.93	10.22	10.86	11.40
PCR	1576.29	1641.95	1712.28	1776.73	1849.83	1899.51	1948.37	2059.96	2155.92
Bacteriologic culture	147.42	152.44	158.71	164.75	171.37	176.03	181.00	191.94	201.34

Abbreviations: ER, emergency room; PCR, polymerase chain reaction

Supplement 5

Table S5. Effectiveness of influenza vaccination in the Northern Hemisphere

Effectiveness	95% CI	Source
**2009–2010**		
56%	23%–75%	Griffin, M.R.; Monto, A.S.; Belongia, E.A.; Treanor, J.J.; Chen, Q.; Chen, J.; Talbot, H.K.; Ohmit, S.E.; Coleman, L.A.; Lofthus, G.; et al. Effectiveness of non-adjuvanted pandemic influenza a vaccines for preventing pandemic influenza acute respiratory illness visits in 4 U. S. Communities. *PLoS One.* **2011**, 6, e23085.
**2010–2011**		
60%	53%–66%	Treanor, J.J.; Talbot, H.K.; Ohmit, S.E.; Coleman, L.A.; Thompson, M.G.; Cheng, P.Y.; Petrie, J.G.; Lofthus, G.; Meece, J.K.; Williams, J.V.; et al. Effectiveness of seasonal influenza vaccines in the United States during a season with circulation of all three vaccine strains. *Clin. Infect. Dis.* **2012**, *55*, 951–959.
**2011–2012**		
47%	36%–56%	Ohmit, S.E.; Thompson, M.G.; Petrie, J.G.; Thaker, S.N.; Jackson, M.L.; Belongia, E.A.; Zimmerman, R.K.; Gaglani, M.; Lamerato, L.; Spencer, S.M.; et al. Influenza vaccine effectiveness in the 2011-2012 season: Protection against each circulating virus and the effect of prior vaccination on estimates. *Clin. Infect. Dis.* **2015**, *58*, 319–327.
59%	43%–70%	Skowronski, D.M.; Janjua, N.Z.; Sabaiduc, S.; De Serres, G.; Winter, A.L.; Gubbay, J.B.; Dickinson, J.A.; Fonseca, K.; Charest, H.; Bastien, N.; et al. Influenza A/subtype and B/lineage effectiveness estimates for the 2011-2012 trivalent vaccine: Cross-season and cross-lineage protection with unchanged vaccine. *J. Infect. Dis.* **2014**, *210*, 126–137.
A(H1N1)pdm09: 42.80%<br>A(H3N2): 38.1%	6.3%–65.0%	Rondy, M.; Castilla, J.; Launay, O.; Costanzo, S.; Ezpeleta, C.; Galtier, F.; de Gaetano Donati, K.; Moren, A. Moderate influenza vaccine effectiveness against hospitalisation with A(H3N2) and A(H1N1) influenza in 2013–14: Results from the InNHOVE network. *Hum. Vaccin. Immunother.* **2016**, *12*, 1217–1224.
**2012–2013**		
49%	43%–55%	McLean, H.Q.; Thompson, M.G.; Sundaram, M.E.; Kieke, B.A.; Gaglani, M.; Murthy, K.; Piedra, P.A.; Zimmerman, R.K.; Nowalk, M.P.; Raviotta, J.M.; et al. Influenza vaccine effectiveness in the United States during 2012-2013: Variable protection by age and virus type. *J. Infect. Dis.* **2015**, *211*, 1529–1540.
A(H3N2): 42.2%<br>A(H1N1)pdm09: 50.4%<br>B: 49.3%	14.9%–60.7%<br>28.4%–65.6%<br>28.4%–65.6%	Kissling, E.; Valenciano, M.; Buchholz, U.; Larrauri, A.; Cohen, J.M.; Nunes, B.; Rogalska, J.; Pitigoi, D.; Paradowska-Stankiewicz, I.; Reuss, A.; et al. Influenza vaccine effectiveness estimates in Europe in a season with three influenza type/subtypes circulating: the I-MOVE multicentre case-control study, influenza season 2012/13. *Euro. Surveill.* **2014**, *19*, pii=20701.

**2013–2014**		
68%	58%–76%	Skowronski, D.M.; Chambers, C.; Sabaiduc, S.; De Serres, G.; Winter, A.L.; Dickinson, J.A.; Gubbay, J.; Fonseca, K.; Charest, H.; Krajden, M.; et al. Integrated sentinel surveillance linking genetic, antigenic, andepidemiologic monitoring of influenza vaccine-virus relatedness and effectiveness during the 2013-2014 influenza season. J. *Infect. Dis.* **2015**, *212*, 726–739.
54%	46%–61%	Gaglani, M.; Pruszynski, J.; Murthy, K.; Clipper, L.; Robertson, A.; Reis, M.; Chung, J.R.; Piedra, P.A.; Avadhanula, V.; Nowalk, M.P.; et al. Influenza vaccine effectiveness against 2009 pandemic influenza A(H1N1) virus differed by vaccine type during 2013-2014 in the United States.
**2014–2015**		
19%	10%–27%	Zimmerman, R.K.; Nowalk, M.P.; Chung, J.; Jackson, M.L.; Jackson, L.A.; Pietre, J.G.; Monto, A.S.; McLean, H.Q.; Belongia, E.A.; Gaglani, M.; et al. 2014-2015 influenza vaccine effectiveness in the United States by vaccine type. *Clin. Infect. Dis.* **2016**, *63*, 1564–1573.
A(H3N2): 14.4%<br>A(H1N1)pdm09: 54.2%<br>B: 48.0%	6.3%–31%<br>31.2%–69.6%<br>28.9%–61.9%	Valenciano, M.; Kissling, E.; Reuss, A.; Rizzo, C.; Gherasim, A.; Horvath, J.K.; Domegan, L.; Pitigoi, D.; Machado, A.; Paradowska-Stankiewicz, I.A.; et al. Vaccine effectiveness in preventing laboratory-confirmed influenza in primary care patients in a season of co-circulation of influenza A(H1N1)pdm09, B and drifted A(H3N2), I-MOVE multicentre case–control study, Europe 2014/15. *Euro. Surveill.* **2016**, *21*, pii=30139.
22%	8%–33%	Puig-Barberà, J.; Burtseva, E.; Yu, H.; Cowling, B.J.; Badur, S.; Kyncl, J.; Sominina, A.; GIHSN. Influenza epidemiology and influenza vaccine effectiveness during the 2014-2015 season: annual report from the Global Influenza Hospital Surveillance Network. *BMC Public Health.* **2016**, *16(Suppl 1)*, 757.
**2015–2016**		
48%	41%–55%	Jackson, M.L.; Chung, J.R.; Jackson, L.A.; Phillips, C.H.; Benoit, J.; Monto, A.S.; Martin, E.T.; Belongia, E.A.; McLean, H.Q.; Gaglani, M.; et al. Influenza vaccine effectiveness in the United States during the 2015-2016 season. *N. Engl. J. Med.* **2017**, *377*, 534 –543.
64%	44%–77%	Chambers, C.; Skowronski, D.M.; Sabaiduc, S.; Winter, A.L.; Dickinson, J.A.; De Serres, G.; Gubbay, J.B.; Drews, S.J.; Martineau, C.; Eshaghi, A.; et al. Interim estimates of 2015/16 vaccine effectiveness against influenza A(H1N1)pdm09, Canada, February 2016. *Euro. Surveill.* **2016**, *21*, 30168.
32.9%	15.5%–46.7%	Kissling, E.; Valenciano, M.; Pozo, F.; Vilcu, A.M.; Reuss, A.; Rizzo, C.; Larrauri, A.; Horvath, J.K.; Brytting, M.; Domegan, L.; et al. 2015/16 I-MOVE/I-MOVE+ multicentre case-control study in Europe: Moderate vaccine effectiveness estimates against influenza A(H1N1)pdm09 and low estimates against lineage-mismatched influenza B among children. *Influenza Other Respir. Viruses.* **2018**, *12*, 423–437.
**2016–2017**		

48%	37%–57%	Flannery, B.; Chung, J.R.; Thaker, S.N.; Monto, A.S.; Martin, E.T.; Belongia, E.A.; McLean, H.Q.; Gaglani, M.; Murthy, K.; Zimmerman, R.K.; et al. Interim estimates of 2016–17 seasonal influenza vaccine effectiveness — United States, February 2017. *MMWR Morb. Mortal. Wkly. Rep.* **2017**, *66*, 167–171.
**2017–2018**		
36%	27%–44%	Flannery, B.; Chung, J.R.; Thaker, S.N.; Monto, A.S.; Martin, E.T.; Belongia, E.A.; McLean, H.Q.; Gaglani, M.; Murthy, K.; Zimmerman, R.K.; et al. Interim estimates of 2016–17 seasonal influenza vaccine effectiveness — United States, February 2017. *MMWR Morb. Mortal. Wkly. Rep.* **2017**, *66*, 167–171.
42%	25%–55%	Skowronski, D.M.; Chambers, C.; De Serres, G.; Dickinson, J.A.; Winter, A.L.; Hickman, R.; Chan, T.; Jassem, A.N.; Drews, S.J.; Charest, H.; et al. Early season co-circulation of influenza A(H3N2) and B(Yamagata): interim estimates of 2017/18 vaccine effectiveness, Canada, January 2018. *Euro. Surveill.* **2018**, *23*, 1–7.
**2018–2019**		
47%	34%–57%	Doyle, J.D.; Chung, J.R.; Kim, S.S.; Gaglani, M.; Raiyani, C.; Zimmerman, R.K.; Nowalk, M.P.; Jackson, M.L.; Jackson, L.A.; Monto, A.S.; et al. Interim estimates of 2018–19 seasonal influenza vaccine effectiveness — United States, February 2019. MMWR *Morb. Mortal. Wkly. Rep.* **2019**, *68*, 135–139.

Abbreviation: CI, confidence interval.

Supplement 6

Table S6.1. Estimated influenza cases averted by immunizing the target population[1]

Scenario	Cases With No Influenza Immunization	Cases With Influenza Immunization 50% Coverage 50% Effectiveness	Cases Averted
0	531,700	398,762	132,939
1	133,868	100,398	33,471
2	267	201	67
4	71,534	53,649	17,885
5	38,575	28,930	9645
6	21,973	16,479	5494
Total	797,918	598,418	199,500

[1] Population aged 50 to 59 years without risk factors for influenza complications.

Table S6.2. Deaths averted by immunizing the target population[1]

Scenario	No Influenza	Influenza Im-	Deaths Averted

	Immunization	munization	
3	0.9	0.7	0.2
7	47.5	35.6	11.9
TOTAL	48.4	36.3	12.1

[1] Population aged 50 to 59 years without risk factors for influenza complications.

Table S6.3. Total economic costs of influenza treatment if the target population[1] is immunized

Scenario	0	1	2	3	4	5	6	7	Cost
Direct costs									
Laboratory diagnosis	.	1.16	0.00	0.0000	6.20	3.66	2.08	0.00	13.11
Medical consultations	.	4.77	0.02	0.0001	3.41	2.93	2.29	0.01	13.42
Drugs	1.04	0.62	0.00	0.0000	0.33	0.20	0.11	0.00	2.30
Hospitalizations	.	.	0.05	0.0013	24.24	54.25	46.35	0.07	124.95
Total direct costs	1.04	6.55	0.07	0.00	34.18	61.02	50.83	0.08	153.78
Indirect costs									
Productivity loss	0.00	5.09	0.03	.	4.53	7.33	7.23	.	24.20
Premature death	.	.	.	0.06	.	.	.	2.91	2.97
Total indirect costs	0.00	5.09	0.03	0.06	4.53	7.33	7.23	2.91	27.17
Total costs of influenza	1.04	11.64	0.10	0.06	38.71	68.35	58.06	2.99	180.95

Assumes 50% coverage and 50% effectiveness.

Amounts are in million US dollars.

[1] Population aged 50 to 59 years without risk factors for influenza complications.

Supplement 7

Figure S1. Projection of population aged 50–59 years and total population in Mexico (2010–2030)

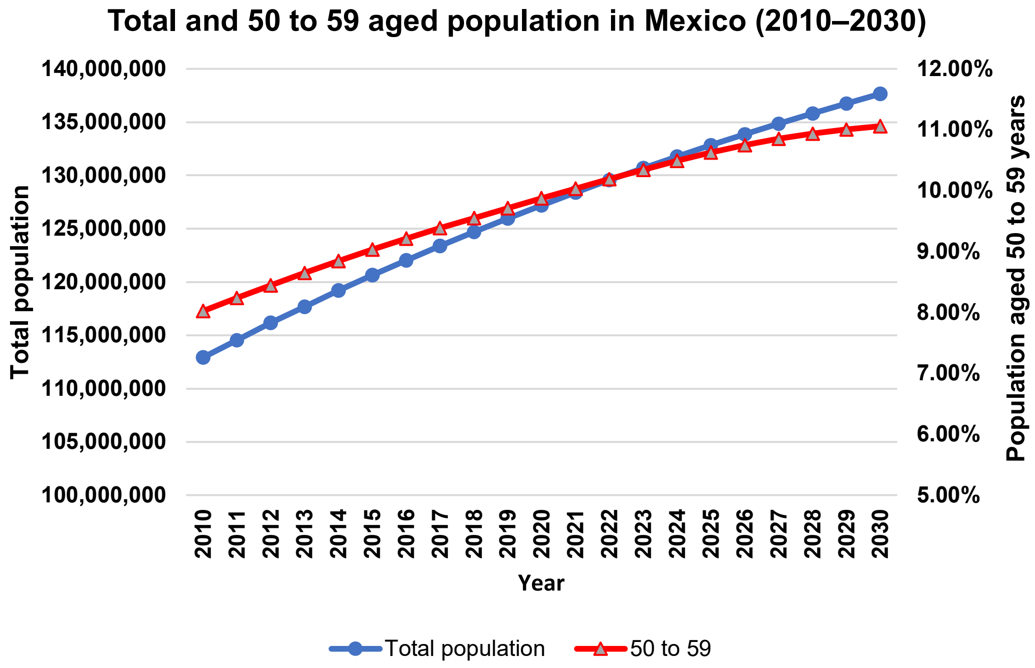

Source: Population projections from Consejo Nacional de Población [National Population Council]

Supplement 8

Text S3. Cases, deaths, and lethality by viral type (2009–2018)

The number of cases, deaths, and lethality of influenza by virus type and age group are shown in **Table S7.2**. For influenza A H1N1, the population group aged 50 to 59 years registered 23% more cases (3277), slightly more deaths (700), and a high lethality rate (21.36%) compared with the ≥60 years age group which registered 2659 cases and 676 deaths (lethality, 25.42%). It is important to note that the population aged ≥60 years is already a target group in the current vaccination schedule, whereas the 50 to 59 age group is not.

Table S7.1. Confirmed influenza cases by age group and season

Age Group, Years	2009–2010	2010–2011	2011–2012	2012–2013	2013–2014	2014–2015	2015–2016	2016–2017	2017–2018	2018–2019	TOTAL	%
**0–4**	382	663	758	543	949	441	1150	899	695	37	**6517**	**12.80**
**5–11**	259	589	756	454	655	309	931	660	466	42	**5121**	**10.06**
**12–17**	205	317	562	279	461	223	507	315	221	9	**3099**	**6.09**
**18–49**	1562	2057	3733	1558	4605	1360	4468	2621	1823	89	**23,876**	**46.91**
**50–59 without risk factors**	155	200	412	177	623	170	582	384	216	16	2935	**5.76**
**50–59 with risk factors**	129	105	310	130	605	164	627	455	247	18	2790	**5.48**
**≥60**	201	398	513	378	1096	575	1370	1135	857	39	**6562**	**12.89**
**TOTAL**	**2893**	**4329**	**7044**	**3519**	**8994**	**3242**	**9635**	**6469**	**4525**	**250**	**50,900**	**100.00**

Source: SISVEFLU

Table S7.2. Cases, deaths, and lethality by viral type (2009–2018)

	A H1N1			A H3N2			A Undetermined			B Yamagata			B Victoria			B Undetermined		
**Age Group, Years**	**Cases**	**Deaths**	**Lethality**	**Cases**	**Deaths**	**Lethality**	**Cases**	**Deaths**	**Lethality**	**Cases**	**Deaths**	**Lethality**	**Cases**	**Deaths**	**Lethality**	**Cases**	**Deaths**	**Lethality**
**<1[1]**	848	65	7.67%	502	10	1.99%	246	10	4.07%	72	1	1.39%	51	3	5.88%	257	14	5.45%
**1–4[1]**	2006	76	3.79%	1323	11	0.83%	465	5	1.08%	158	4	2.53%	144	1	0.69%	445	4	0.90%
**5–11**	2057	58	2.82%	1327	9	0.68%	373	0	0%	280	2	0.71%	241	0	0%	843	6	0.71%
**12–19**	1666	44	2.64%	1146	5	0.44%	287	2	0.70%	230	0	0%	185	2	1.08%	544	11	2.02%
**20–24**	1731	54	3.12%	993	8	0.81%	288	3	1.04%	71	3	4.23%	119	1	0.84%	280	3	1.07%
**25–29**	2180	113	5.18%	1101	13	1.18%	343	10	2.92%	76	1	1.32%	119	0	0.00%	345	5	1.45%
**30–34**	2074	150	7.23%	1181	5	0.42%	383	7	1.83%	106	0	0%	102	2	1.96%	340	3	0.88%
**35–39**	2090	227	10.86%	922	12	1.30%	367	10	2.72%	154	5	3.25%	51	0	0%	316	5	1.58%
**40–44**	2136	314	14.70%	865	21	2.43%	294	19	6.46%	171	3	1.75%	23	0	0%	304	8	2.63%
**45–49**	1893	330	17.43%	754	23	3.05%	253	20	7.91%	163	6	3.68%	25	0	0%	304	15	4.93%
**50–54 without risk factors**	912	96	10.53%	351	6	1.71%	133	6	4.51%	71	2	2.82%	10	0	0%	152	3	1.97%
**50–54 with risk factors**	837	227	27.12%	308	25	8.12%	102	7	0%	51	1	1.96%	13	0	0%	110	10	9.09%
**55–59 without risk factors**	721	129	17.89%	313	8	2.56%	96	3	3.13%	60	2	3.33%	8	0	0%	108	5	4.63%
**55–59 with risk factors**	807	248	30.73%	286	20	6.99%	75	14	0%	63	4	6.35%	12	1	8.33%	126	16	12.70%
**60–64[1]**	1022	275	26.91%	489	47	9.61%	116	18	15.52%	51	2	3.92%	11	0	0%	165	18	10.91%
**65–69[1]**	603	162	26.87%	369	41	11.11%	95	9	9.47%	66	6	9.09%	22	2	9.09%	114	26	22.81%
**70–74[1]**	392	103	26.28%	378	43	11.38%	79	7	8.86%	43	6	13.95%	15	1	6.67%	114	10	8.77%
**75–79[1]**	293	58	19.80%	414	47	11.35%	69	7	10.14%	45	4	8.89%	7	1	14.29%	101	16	15.84%
**80–84[1]**	194	45	23.20%	330	43	13.03%	59	3	5.08%	33	2	6.06%	4	1	25.00%	66	13	19.70%
**85–89[1]**	107	25	23.36%	266	39	14.66%	36	3	8.33%	17	2	11.76%	3	0	0%	55	5	9.09%
**90–94[1]**	35	6	17.14%	143	14	9.79%	18	2	11.11%	8	1	12.50%	5	1	20.00%	28	11	39.29%
**≥95[1]**	13	2	15.38%	43	7	16.28%	11	4	36.36%	4	0	0%	2	0	0%	9	4	44.44%
**TOTAL**	**24,617**	**2807**	**11.40%**	**13,804**	**457**	**3.31%**	**4188**	**169**	**4.04%**	**1993**	**57**	**2.86%**	**1172**	**16**	**1.37%**	**5126**	**211**	**4.12%**

[1] Target groups for seasonal influenza vaccination, according to Mexican guidelines.

Table S7.3. Estimated influenza cases by age group and season

Age Group, Years	2010–2011	2011–2012	2012–2013	2013–2014	2014–2015	2015–2016	2016–2017	2017–2018	2018–2019	TOTAL
**0–4**	1,541,921	526,212	1,991,434	1,417,562	1,794,872	1,275,233	1,268,274	2,197,168	1,476,442	**13,489,116**
**5–11**	1,291,012	583,585	1,952,068	1,164,843	1,865,513	1,266,616	1,729,655	2,176,925	1,491,125	**13,521,341**
**12–17**	1,100,268	496,595	1,658,823	989,697	1,586,659	1,080,209	1,480,577	1,869,632	1,283,742	**11,546,202**
**18–49**	2,982,160	1,421,706	4,716,432	5,471,988	3,646,289	4,320,570	3,679,992	6,247,656	4,245,921	**36,732,715**
**50–59**	777,680	312,180	1,308,217	1,445,898	1,267,128	1,259,132	1,581,055	2,928,240	1,515,030	**12,394,559**
**≥60**	582,078	275,863	1,176,698	791,211	1,253,279	727,139	1,335,003	2,329,457	1,211,712	**9,682,439**
TOTAL	**8,275,119**	**3,616,140**	**12,803,672**	**11,281,198**	**11,413,739**	**9,928,898**	**11,074,556**	**17,749,078**	**11,223,972**	**97,366,372**
**Population**	**114,551,762**	**116,179,714**	**117,688,241**	**119,216,240**	**120,653,293**	**122,038,924**	**123,388,002**	**124,692,044**	**125,960,168**	
**Attack rate (%)**	**7.22**	**3.11**	**10.88**	**9.46**	**9.46**	**8.14**	**8.98**	**14.23**	**8.91**	

Table S7.4. Lethality by age group and season

Age Group, Years	2009–2010	2010–2011	2011–2012	2012–2013	2013–2014	2014–2015	2015–2016	2016–2017	2017–2018	2018–2019	Average
**0–4**	1.83%	0.45%	3.43%	1.47%	5.48%	1.36%	4.61%	3.45%	2.16%	8.11%	**3.13%**
**5–11**	1.54%	0.00%	0.93%	0.44%	2.60%	0.97%	2.58%	1.52%	1.72%	0.00%	**1.46%**
**12–17**	1.95%	0.32%	1.25%	0.36%	2.39%	1.79%	1.58%	0.95%	2.26%	0.00%	**1.42%**
**18–49**	7.23%	0.92%	3.16%	1.93%	10.49%	2.94%	6.85%	8.36%	4.61%	7.87%	**5.94%**
**50–59 without risk factors**	5.81%	0.50%	5.10%	1.69%	15.57%	4.71%	9.79%	12.24%	7.41%	6.25%	**8.86%**
**50–59 with risk factors**	20.16%	5.71%	17.74%	6.15%	28.26%	9.76%	24.88%	19.56%	16.60%	27.78%	**20.54%**
**≥60**	9.45%	6.28%	18.32%	7.14%	23.72%	13.39%	19.05%	22.29%	14.47%	5.13%	**17.40%**
**TOTAL**	6.29%	1.27%	4.66%	2.24%	12.13%	4.75%	8.98%	10.08%	6.48%	7.20%	**7.30%**

Source: SISVEFLU

Table S7.5. Years of life lost per age group by season

Age Group, Years	2009–2010	2010–2011	2011–2012	2012–2013	2013–2014	2014–2015	2015–2016	2016–2017	2017–2018	2018–2019	Total	%
**0–4**	366	151	1847	604	2831	458	3053	1408	1053	154	**11,925**	12.47%
**5–11**	284	0	460	132	1149	207	1656	664	549	0	**5101**	5.33%
**12–17**	236	62	431	61	685	240	494	186	314	0	**2709**	2.83%
**18–49**	4425	709	4479	1140	17,292	1596	10,715	7747	3128	247	**51,478**	53.84%
**50–59**	747	131	1498	215	5388	529	4319	2785	1198	131	**16,941**	17.72%
**≥60**	154	115	716	144	1960	372	1697	1682	617	9	**7466**	7.81%
**TOTAL**	**6212**	**1168**	**9431**	**2296**	**29,305**	**3402**	**21,934**	**14,472**	**6859**	**541**	**95,620**	100.00%

Source: SISVEFLU
